# Activation of Cerebellum, Basal Ganglia and Thalamus During Observation and Execution of Mouth, hand, and foot Actions

**DOI:** 10.1007/s10548-023-00960-1

**Published:** 2023-05-03

**Authors:** Antonino Errante, Marzio Gerbella, Gloria P. Mingolla, Leonardo Fogassi

**Affiliations:** 1 Department of Medicine and Surgery, University of Parma, Via Volturno 39, 43125 Parma, Italy; 2 Department of Diagnostics, Neuroradiology unit, University Hospital of Parma, Via Gramsci 14, 43126 Parma, Italy; 3 Department of Neurosciences, Biomedicine and Movement Sciences, University of Verona, Piazzale Ludovico Antonio Scuro 10, 37124 Verona, Italy

**Keywords:** Action observation, Mirror neuron system, Somatotopy, fMRI, cerebellum, Basal ganglia, Thalamus

## Abstract

Humans and monkey studies showed that specific sectors of cerebellum and basal ganglia activate not only during execution but also during observation of hand actions. However, it is unknown whether, and how, these structures are engaged during the observation of actions performed by effectors different from the hand. To address this issue, in the present fMRI study, healthy human participants were required to execute or to observe grasping acts performed with different effectors, namely mouth, hand, and foot. As control, participants executed and observed simple movements performed with the same effectors. The results show that: (1) execution of goal-directed actions elicited somatotopically organized activations not only in the cerebral cortex but also in the cerebellum, basal ganglia, and thalamus; (2) action observation evoked cortical, cerebellar and subcortical activations, lacking a clear somatotopic organization; (3) in the territories displaying shared activations between execution and observation, a rough somatotopy could be revealed in both cortical, cerebellar and subcortical structures. The present study confirms previous findings that action observation, beyond the cerebral cortex, also activates specific sectors of cerebellum and subcortical structures and it shows, for the first time, that these latter are engaged not only during hand actions observation but also during the observation of mouth and foot actions. We suggest that each of the activated structures processes specific aspects of the observed action, such as performing internal simulation (cerebellum) or recruiting/inhibiting the overt execution of the observed action (basal ganglia and sensory-motor thalamus).

**Supplementary Information:**

The online version contains supplementary material available at 10.1007/s10548-023-00960-1.

## Introduction

It is well known that a particular class of monkey cortical visuomotor neurons, named “mirror neurons”, are activated during the execution as well as the observation of motor acts performed by another individual (di Pellegrino et al. 1992; Gallese et al. 1996). It has been proposed that, thanks to this visuomotor matching mechanism, an observed motor act produces in the observer’s brain a motor activation as if the observer was actually executing it (Rizzolatti et al. 2014). Mirror neurons were originally described in monkey ventral premotor area F5 and in inferior parietal area PFG (di Pellegrino et al. 1992; Gallese et al. 1996; Fogassi et al. 2005; Rozzi et al. 2008; Rizzolatti and Fogassi 2014), but studies of the last few years provided evidence of the presence of neurons with mirror properties in a more extended network of cortical areas (Tkach et al. 2007; Dushanova and Donoghue 2010; Rizzolatti et al. 2014; Pani et al. 2014; Maeda et al. 2015; Falcone et al. 2016; Lanzilotto et al. 2016, 2019; Simone et al. 2017; Papadourakis and Raos 2019). Electrophysiological (EEG, MEG, TMS) and brain imaging (fMRI, PET) studies demonstrated that a system with mirror properties (Mirror Neuron System, MNS) is also present in humans (Fadiga et al. 1995; Cochin et al. 1998; Hari et al. 1998; Cochin et al. 1999; Gazzola and Keysers 2009; Caspers et al. 2010; Molenberghs et al. 2012), (for review, see Rizzolatti et al. 2014).

Although no monkey study did provide any direct electrophysiological evidence that the MNS involves also the cerebellum and subcortical structures, neuroanatomical data showed that specific sectors of the cerebellum, striatum, and thalamus are connected with cortical areas endowed with mirror properties (Glickstein et al. 1985; Schmahmann and Pandya 1989; Middleton and Strick 1997; 2000; Clower et al. 2005; Gerbella et al. 2016; Bostan and Strick 2018; Bruni et al. 2018), raising the question of the presence of neurons with these properties in cerebellar and subcortical territories with well-known motor characteristics. A recent monkey study employing 14 C-deoxyglucose autoradiographic method (Raos and Savaki 2021) showed that specific parts of the cerebellum are activated both during observation and execution of grasping actions. Furthermore, electrophysiological and fMRI human studies showed that both execution and observation of hand motor acts activate specific sectors of the cerebellum, plus the basal ganglia (Gazzola and Keysers 2009; Alegre et al. 2010; Casiraghi et al. 2019; Abdelgabar et al. 2019; Errante and Fogassi 2020).

Monkey studies showed, in both premotor and parietal cortex, the presence of mirror neurons activated by observation of hand and mouth motor acts (Gallese et al. 1996; Ferrari et al. 2003; Rozzi et al. 2008). Human experiments with fMRI technique showed that the parieto-frontal MNS also involves foot actions, and it is somatotopically organized, suggesting that when an individual observes a motor act performed with a specific effector, an internal replica of that motor act is automatically generated, in terms of effector specificity (Buccino et al. 2001; Gazzola and Keysers 2009). In a subsequent work, Jastorff and colleagues (2010), on the one hand, confirmed the somatotopic activation of premotor cortex when subjects observed several actions performed with mouth, hand, and foot, on the other hand, they showed that the parietal activation is related to the type of action, such as grasping, even when this latter is performed by other effectors.

Differently from the data reported for cortical areas, there are no studies addressing whether, and how, cerebellum and subcortical structures are engaged when the observed actions are executed by the observed agent using effectors different from the hand, such as mouth and foot. In this respect, the presence of a motor somatotopy in monkey and human cerebellum (Snider and Eldred 1952; Grodd et al. 2001; Schlerf et al. 2010; Boillat et al. 2020) as well as in monkey basal ganglia and thalamus (Alexander et al. 1986; Vitek et al. 1994, 1996) allows one to hypothesize that a somatotopic organization can also be present during action observation. In order to test this hypothesis, we carried out an fMRI study in which healthy human participants were instructed to execute or to observe grasping acts or simple movements, as control, performed with different effectors, namely mouth, hand, and foot. The results showed that cerebellum, basal ganglia and thalamus are engaged not only during observation of hand actions but also during observation of mouth and foot actions. The analysis of the activations shared by the observation and execution conditions indicates that, similarly to the cortex, the territories of cerebellum and subcortical structures activated during the execution and observation of goal-related actions are somatotopically organized.

## Materials and Methods

### Participants

Eighteen human volunteers were involved in the study (8 females; mean age = 25.6 years; SD = 3.9; range = 21–35 years). All subjects were students of the University of Parma. They had normal or corrected-to-normal vision and were reimbursed for their participation. Only participants with no history of neurological, orthopedic, or rheumatological disorders, and no drug or alcohol abuse, were recruited. All participants were right-handed according to the Edinburgh Handedness Inventory (Oldfield 1971). Movements during scanning motion were detected based on the three translation and rotation parameters resulting from 3D motion correction (cut-off criterion: <3 mm for translation, < 3 degree for rotation). Two participants were subsequently excluded from data analysis because they presented excessive head motion. To summarize, a total of 16 participants (8 females; mean age = 24.8 years; SD = 3.6; range = 21–35 years) were included in the subsequent analyses.

The sample size was based on the calculation of the change in the Blood Oxygenation Level Dependent (BOLD) signal detected in a previous (not published) fMRI study with an action observation/execution paradigm carried out in 22 participants. In this latter study, significant activation clusters within the MNS cortical areas, including the PMv and the IPL, and other clusters including putamen and cerebellum (lobule VI) were considered for a power analysis, using a statistical threshold of *p* < 0.001 with Family Wise Error (FWE) correction for multiple comparisons at the cluster level. This analysis (software: NeuroPower) showed that to obtain a change in the BOLD signal with respect to the baseline condition, with a significance threshold of *p* < 0.05 and a minimum statistical power of 80% with Bonferroni correction, it is necessary to recruit at least 14 participants.

#### Ethical Approval

Written informed consent was obtained from all individual participants included in the study. The study was approved by the local ethics committee (Comitato Etico per Parma, University of Parma; code UNIPRMR750v1). All procedures were carried out in accordance with The Code of Ethics of the World Medical Association (Declaration of Helsinki).

### General Experimental Procedure

Before starting the imaging session, participants were trained to perform the action execution task outside the MR scanner (training) and practiced it also within the scanner (for about 15 min) just before starting the scanning. During the training, participants became familiar with the stimuli and received instructions. Only when the subject reached 100% of accuracy during the training period, the experimental session could start.

**Fig. 1:**
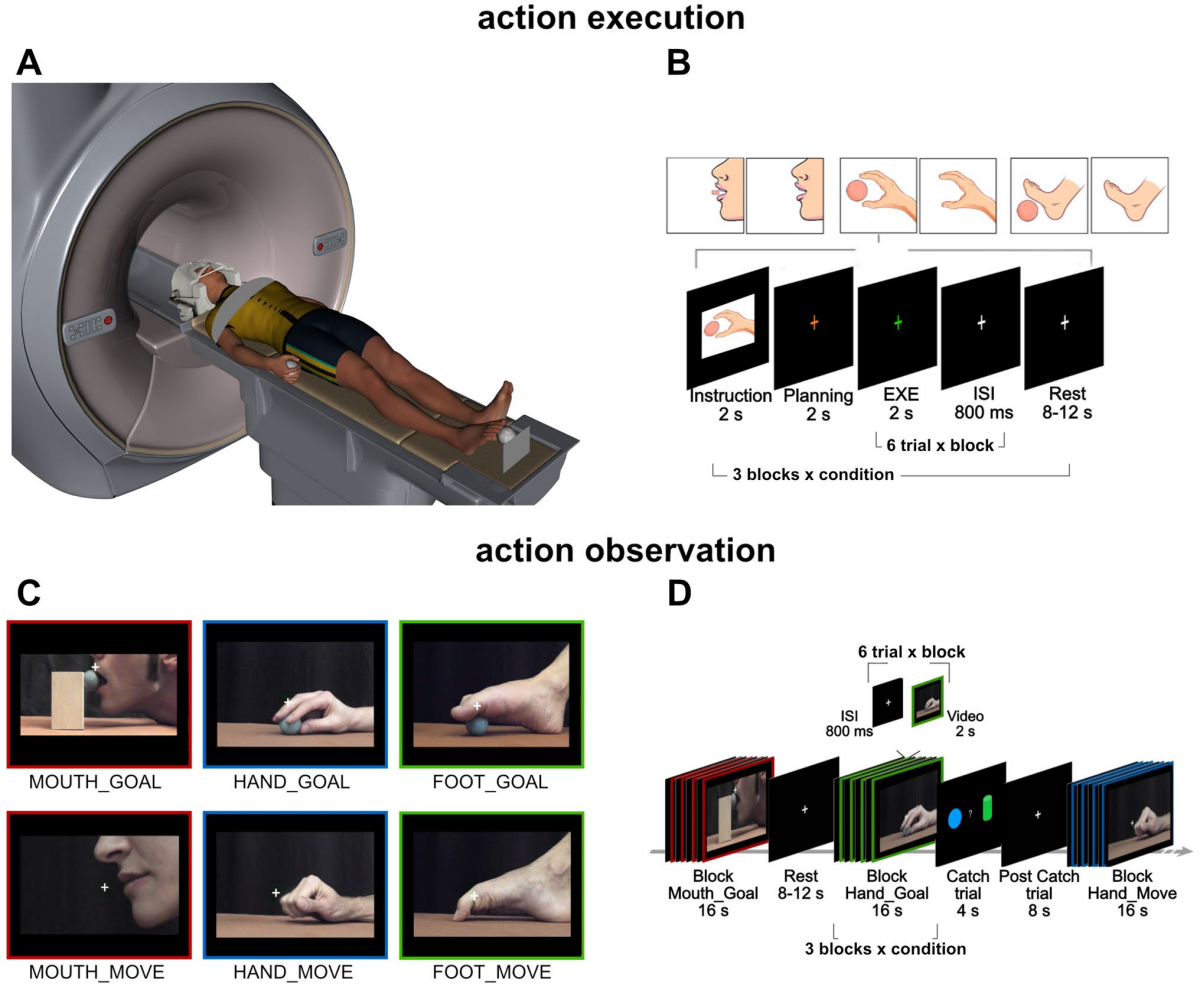
Experimental design, stimuli, and tasks.** (A)** Illustration of the experimental setting during the action execution and observation tasks. **(B)** Action execution task performed in two runs, with mixed block-event-related paradigm. Each run included three blocks for condition, and each block was composed by six motor trials belonging to the same condition. **(C)** Static frames taken from the video stimuli used for the action observation task. Each frame represents the time in which the effector grasps the objects, in the goal-directed grasping conditions, or the end of the closing act in the case of simple movement conditions. **(D)** Action observation paradigm, made by independent blocks, each composed by six videos of the same condition, interspersed with the rest period

They lay supine in the bore of the scanner in a dimly lit environment (Fig. 1A). Visual instructions and stimuli were presented using a digital goggles system (Resonance Technology, Northridge, CA) (60 Hz refresh rate) with a resolution of 800 horizontal pixels x 600 vertical pixels, with a horizontal eye field of 30°. Digital signal transmission to the scanner was via optic fiber. MR-compatible headphones were used to give instructions to the subjects and to muffle the scanner noise. Software E-Prime 2 Professional (Psychology Software Tools, Inc.; http://www.pstnet.com) was used for stimuli presentation.

No participant reported any difficulty in performing the tasks. During scanning, subject’s performance was visually checked by the experimenter by on-line video screening using an MR-compatible camera, in order to verify the correctness of trial execution.

### Experimental Design

The experiment consisted in four runs, subdivided in two tasks: (a) action execution (run 1–2), performed without vision, in which participants were required to perform grasping actions with the mouth, the hand, or the foot and, as control, a simple motor task consisting of opening-closing the mouth, the hand or the foot (Fig. 1B); (b) action observation (run 3–4), in which participants observed passively video stimuli showing the same type of actions performed in the execution task (Fig. 1C and D). The presentation order of the observation/execution runs was counterbalanced across participants; namely, half of the participants started with the observation runs, followed by the action execution runs (Obs-Exe), while the remaining participants started with the action execution runs (Exe-Obs).

### Stimuli

#### Action Execution

The action execution task was carried out by presenting subjects with MR-compatible 3D objects. During hand actions, a sphere (40 mm diameter) was placed medially to the hand at a distance of 1.5 cm from the thumb (Fig. 1A). During execution of foot trials, the object was placed in front and medially with respect to the foot, on a wooden support, at a height of 17.5 cm from the scanner bed. The distance of the object from the big toe was of 1.5 cm. During execution of mouth trials, a bar made of plastic material (20 cm in length, diameter 1 cm) attached to the head coil through a flexible system, was positioned at a distance of 1 cm from the lips to minimize the head movements required to grasp it. The use of the bar, instead of the sphere used for hand and foot actions, allowed participants to perform mouth movements with minimal movement of the lips, avoiding involuntary head movements. Note, however, that the shape of the used object does not change the goal of the action, which remains that of grasping with the mouth.

Video-clips showing an example of the type of movements performed by participants within the scanner are included as Supplementary Information (Suppl. Video 1–6).

#### Action Observation

Visual stimuli consisted of 2 s videos (see Fig. 1C) showing the same types of goal-directed actions or simple movements used in the action execution task, performed by two actors (male, female) with the mouth, the right hand, and the right foot, from a lateral perspective. All videos were recorded by means of a digital HD camera (© GoPro, Inc., USA), with a frame rate of 100/second and a resolution of 1280 × 720p.

The objects used in the goal-directed action videos were 3D geometric solids (a sphere, a cylinder, a cube). A total of 18 video clips were recorded (2 actors x 3 conditions x 3 object types). Concerning simple movements videos, in order to increase the movement variability, three repetitions of the same type of movement were recorded, for each effector. Thus, a total number of 18 simple movement video clips were used (2 actors x 3 conditions x 3 repetitions).

### Experimental Paradigm

#### Action Execution task

Participants laid supine in the scanner. They performed three types of grasping actions: grasping with the mouth (*Exe Mouth Goal*), grasping with the hand (*Exe Hand Goal*), and grasping with the foot (*Exe Foot Goal*). In addition, there were three control conditions consisting in simple opening-closing movements performed with the mouth (*Exe Mouth Move*), the hand (*Exe Hand Move*), or the foot (*Exe Foot Move*). Participants wore digital goggles system and headphones to receive the instructions. Each participant was instructed to place her/his right hand and foot in a comfortable resting position (Fig. 1A) and to maintain fixation, during the entire experiment, on a white cross presented on the center of a black screen. To limit head movements during movement execution, the participant’s head was supported within the MR coil using foam. In addition, the participant’s right forearm was strapped with an elastic band. Throughout the experiment, the participant’s actions were recorded by an MR-compatible camera placed at the entrance of the magnet bore. Concerning goal-related conditions, each action started with the effector close to the target object and terminated in the same starting position. The positioning of the object near the effector allowed the participant to perform grasping actions without additional proximal/axial movements. Concerning simple movements, each opening/closing movement was performed keeping the effector in the same starting position.

Each of the two action execution runs was acquired using a mixed block-event-related paradigm (Fig. 1B), consisting in 18 blocks, three blocks for each of the six conditions. Each block included six motor trials, thus, in the whole experiment, subjects were required to perform 36 motor trials for each condition, 18 trials per run. The task sequence of the execution blocks was as follows. At the beginning of each block, an instruction cue (a drawing representing the effector to be used and the type of required action/movement) was presented for 2 s, then an orange fixation cross appeared in the center of the black screen, in order to take into account movement preparation. After 2 s, the fixation cross color turned to green instructing the subject to perform, without vision, a series of six grasping trials (or six simple movements). The duration of each motor trial was 2 s, cued by the presentation of the green fixation cross. After each trial period, an interstimulus interval (ISI) of 800 ms was presented to separate consecutive motor trials within the block. A total of five ISI was used, because the last video was followed by the rest period. Thus, the duration of each execution block was 20 s, including Instruction (2 s), Planning (2 s) and 6 actions/movements (12 s, 2 s for each trial, plus 5 ISI of 800 ms each, thus 4 s of ISI). In the present study, we focused the analysis on the execution phase, consisting in a block of six motor trials (duration 16 s). Motor blocks were interleaved with the baseline condition (*rest*) consisting of the static presentation of a white cross in the middle of the screen (8, 10, or 12 s). The paradigm was administered alternating grasping blocks, simple movement blocks, and rest periods in a counterbalanced manner among subjects. The duration of each run was about 9 min.

#### Action Observation Task

The action observation task was performed in two separate runs, acquired using a block paradigm (Fig. 1D). A total of six conditions were presented, three types of goal-directed actions and three types of simple movements, as a control. The goal-directed conditions included observation of grasping performed with the mouth (*Obs Mouth Goal*), the hand (*Obs Hand Goal*), or the foot (*Obs Foot Goal*). The three simple movement control conditions included opening-closing movements performed with the mouth (*Obs Mouth Move*), the hand (*Obs Hand Move*), or the foot (*Obs Foot Move*). Each block lasted 16 s and it was composed of 6 consecutive videos of the same condition, interspersed with 5 ISI of 800 ms each (Fig. 1D). During a typical observation run, a total of 18 blocks of stimuli were presented, three blocks for each condition. Each goal-directed and simple movement video was presented 36 times during the experiment, 18 times per run.

The order of blocks (and therefore of conditions) was counterbalanced across subjects. The entire action observation task consisted of a total of 36 blocks, six blocks (corresponding to 36 trials) for each condition. Blocks of stimuli were interleaved by *rest* condition lasting 8, 10, or 12 s, used as baseline, in which participants had to fixate a white cross presented in the middle of a black screen. The duration of each observation run was about 8 min.

#### Control Test for Task Attention

To ensure that participants attended to the visual stimuli, in five blocks per run, after viewing 2, 4, or 6 stimuli in a block, a catch trial was presented, in which they had to provide an explicit response concerning the type of object presented in the last video (for example, cube or sphere?), using a two-button response pad. For each catch-trial, two drawings of possible objects were presented on the screen, together with the question asking participants to indicate the correct response. The catch-trials (lasting 4 s each) were followed by an 8 s rest period to remove movement-related artefacts. For each participant, 10 responses were recorded in the observation runs. The mean response accuracy of participants was 93.1% (*SD* = 4.7).

### Data Acquisition

MR images were acquired with a 3T General Electric scanner (MR750 Discovery) equipped with a 32-channel receiver head-coil. Functional volumes were acquired while participants performed the corresponding action observation task and action execution task, with the following parameters: forty axial slices of functional images covering the whole brain acquired using a gradient-echo echo-planar imaging (EPI) pulse sequence, slice thickness = 3, plus interslice gap = 0.5 mm, 64 × 64 × 37 matrix with a spatial resolution of 3.5 × 3.5 × 3.5 mm, TR = 2000 ms, TE = 30 ms, FOV = 205 × 205 mm^2^, flip angle = 90°, in plane resolution = 3.2 × 3.2 mm^2^. A 3D isotropic T1-weighted-images sequence was acquired as anatomical reference. Its acquisition parameters were as follows: 196 slices, 280 × 280 matrix with a spatial resolution of 1 × 1 × 1 mm, TR = 9700 ms, TE = 4 ms, FOV = 252 × 252 mm; flip angle = 9°.

### FMRI Data Preprocessing

Data processing was performed with SPM12 (Wellcome Department of Imaging Neuroscience, University College, London, UK; http://www.fil.ion.ucl.ac.uk/spm) running on MATLAB R2018a (The Mathworks, Inc.). Structural images were centered and reoriented with functional images with respect to the anterior-posterior commissure axis. The first four EPI volumes of each functional run were discarded to allow the static magnetic field to reach a steady state. For each subject, all volumes were slice timing corrected, spatially realigned to the first volume of the first functional run, and un-warped to correct for between-scan motion. Motion parameters (Supplementary Methods) were used as predictors of no interest in the subsequent statistical analysis (see below) to account for translation and rotation along the three possible dimensions as determined during the realignment procedure. The cut-off used for motion correction tolerance was the size of the voxel (3 mm). If motion exceeded this measure in translation and/or rotation, the dataset was not included in the analysis. Suppl. Figure 1 shows movement parameters during fMRI execution runs. As evident, motion was within 3 mm and no difference in translation/rotation was present between movements performed with the three effectors. The T1-weighted image was segmented into grey, white and cerebrospinal fluid and spatially normalized to the Montreal Neurological Institute (MNI) space. Spatial transformation derived from this segmentation was then applied to the realigned EPIs for normalization and re-sampled in 2 × 2 × 2 mm^3^ voxels using trilinear interpolation in space. All functional volumes were then spatially smoothed with an 8-mm full-width half-maximum isotropic Gaussian kernel (FWHM). For the normalization of cerebellar data, the T1-weighted images were deformed to fit the template of the human cerebellum using SUIT toolbox (Diedrichsen et al. 2009). The toolbox allows to isolate the cerebellum and creates a mask. Non-linear deformation was then applied to each contrast image.

### FMRI Statistical Analysis

Data were analyzed using a random-effects model (Friston et al. 1999), implemented in a two-level procedure. The first level analysis was performed using the whole-brain single-subject fMRI responses, modeled using two different General Linear Models (GLMs), one for the execution and one for the observation task. The design-matrix of the first GLM (action execution) included eight predictors (*Instruction, Planning phase, Exe Mouth Goal, Exe Hand Goal, Exe Foot Goal, Exe Mouth Move, Exe Hand Move, Exe Foot Move*), convolved with the hemodynamic response function (HRF), plus six predictors obtained from the motion correction in the realignment process to account for voxel intensity variations caused by head-movement. The matrix of the second GLM (action observation) included onsets and durations of each experimental condition, and the response to catch-trials (*Obs Mouth Goal, Obs Hand Goal, Obs Foot Goal, Obs Mouth Move, Obs Hand Move, Obs Foot Move*, and *Response*), plus six predictors obtained from motion correction. All regressors, except for *Response*, were composed by six consecutive videos, which were modeled as one single epoch lasting 16 s. Catch trials were modeled as consecutive blocks, lasting 12 s each, including the effective response time (4 s) and a signal-denoising period (8 s) to separate the motor component from subsequent processing. Contrasts derived from parameter estimation were calculated and entered into a flexible factorial within-subjects analysis of variance (ANOVA). Specific effects were tested using *t* statistical parametric maps (SPMt), with degrees of freedom corrected for non-sphericity at each voxel.

At first level, the same models were also tested using the peak-level inference (Penny et al. 2007), in order to assess specific activations of basal ganglia and thalamus during execution and observation tasks. To this aim, the GLMs, including the same regressors of the whole brain analysis described above, were calculated using subcortical explicit Region of Interest (ROI) masks. A first mask included the basal ganglia (bilaterally), in particular: putamen, globus pallidus, caudate nucleus, red nucleus, and subthalamic nucleus. It was selected from the Atlas of the basal Ganglia (ATAG, https://www.nitrc.org/projects/atag/) (Keuken and Forstmann 2015). Concerning the thalamus, the explicit mask was selected from the Atlas of the Thalamus (Thomas Atlas, https://www.lead-dbs.org/helpsupport/knowledge-base/atlasesresources/atlases/) (Su et al. 2019).

At second level, a group-based statistical analysis was performed for each considered map (whole brain, basal ganglia, thalamus), thus resulting in three second-level models. The corresponding t-contrast images of the first-level models were entered in a flexible ANOVA with sphericity correction for repeated measures (Friston et al. 2002). Each second-level model included 12 regressors (*Exe Mouth Goal vs. Rest, Exe Hand Goal vs. Rest, Exe Foot Goal vs. Rest, Exe Mouth Move vs. Rest, Exe Hand Move vs. Rest, Exe Foot Move vs. Rest*, and the corresponding six observation conditions). An additional regressor was included in the analysis in order to control for the effect of the order of presentation of the observation/execution tasks. At this level, we computed the main contrasts between *goal-related* and *simple movements* conditions, for both observation and execution tasks, in order to assess the specific functional organization of the examined areas during the observation and execution of goal-directed actions, characterized by the presence of a target object and by the interaction between the effector and the object.

Contrast images, derived from direct contrasts between goal-related and simple movement conditions (e.g., *Obs Mouth Goal* vs. *Obs Mouth Move* AND *Exe Mouth Goal* vs. *Exe Mouth Move*) were entered in the subsequent *conjunction analysis* (Friston et al. 2005), performed to highlight cortical, cerebellar, basal ganglia and thalamus regions involved in both execution and observation tasks. In particular, we calculated three types of conjunction maps (whole-brain, basal ganglia, and thalamus) for each effector (Mouth, Hand, Foot). All these conjunctions were formulated as *conjunction null hypotheses* and therefore only yield activations that are significantly present in both original contrasts of the conjunction (also referred to as minimum statistics). That is, conjunctions represent a logical “and”, requiring both contrasts to be separately significant for the conjunction to be significant. Additionally, overlap maps on the cerebral cortex, cerebellum, basal ganglia, and thalamus were created using the clusters obtained with the conjunction analysis.

Statistical inference was drawn, except when stated, at the cluster level, with a threshold of *p* < 0.001 corrected for multiple comparisons using Family-Wise Error correction (FWE). Local maxima of activations are presented in the stereotaxic space of the MNI coordinate system. Activations were also localized using cytoarchitectonic probabilistic maps of the human brain, using the SPM-Anatomy toolbox v3.0 (Eickhoff et al. 2005).

Finally, the center of gravity (COG) for each effector was computed using single-subject first-level results obtained from models run on cerebellum, basal ganglia, and thalamus, respectively. Within each effector-specific shared representation derived from the conjunction analysis, the coordinates of the peak activation (maximum *t*-value) were extracted and transformed into MNI coordinates. In addition, for each region, Euclidean 3D distances between the COGs were computed by d_(E1, E2)_=$\sqrt{{(\text{E}1xi-\text{E}2xi)}^{2}+{(\text{E}1yi-\text{E}2yi)}^{2}+{(\text{E}1zi-\text{E}2zi)}^{2}}$ with x*i*, y*i*, and z*i* being medial-lateral, rostro-caudal, and dorso-ventral MNI peak coordinates, and E1, E2 are the considered effectors. Overall, for each region, the following distances between effectors were computed: Mouth vs. Hand, Mouth vs. Foot, and Foot vs. Hand. These distances represent the shortest path in 3D space. In order to confirm the presence of a significant distance between effectors representation, a one-sample *t-test* against 0 was performed.

To further assess the presence of a somatotopic organization, a Principal Component Analysis (PCA) was computed to determine the main axis of orientation (corresponding to the first and second principal components) along the MNI coordinates of peak activations of the three effectors representations (mouth, hand, foot) within each structure (cerebellum, basal ganglia, thalamus) and for each individual subject. The PCA procedure allows the spatial remapping of the MNI coordinates along the axis explaining most of the variance.

## Results

In the following paragraphs, we describe the network of cerebral structures activated during the execution and observation of *goal-related* grasping actions performed with mouth, hand, and foot. In order to show the specific activations due to these latter conditions, we performed a direct comparison with the observation/execution of *simple movements* performed with the same effectors. No significant activations were associated with the order of presentation of the action observation/execution tasks. A complete description of the activations corresponding to each condition vs. *rest* is reported in Supplementary Information.

### Brain Activations During Execution of Grasping Actions

**Fig. 2:**
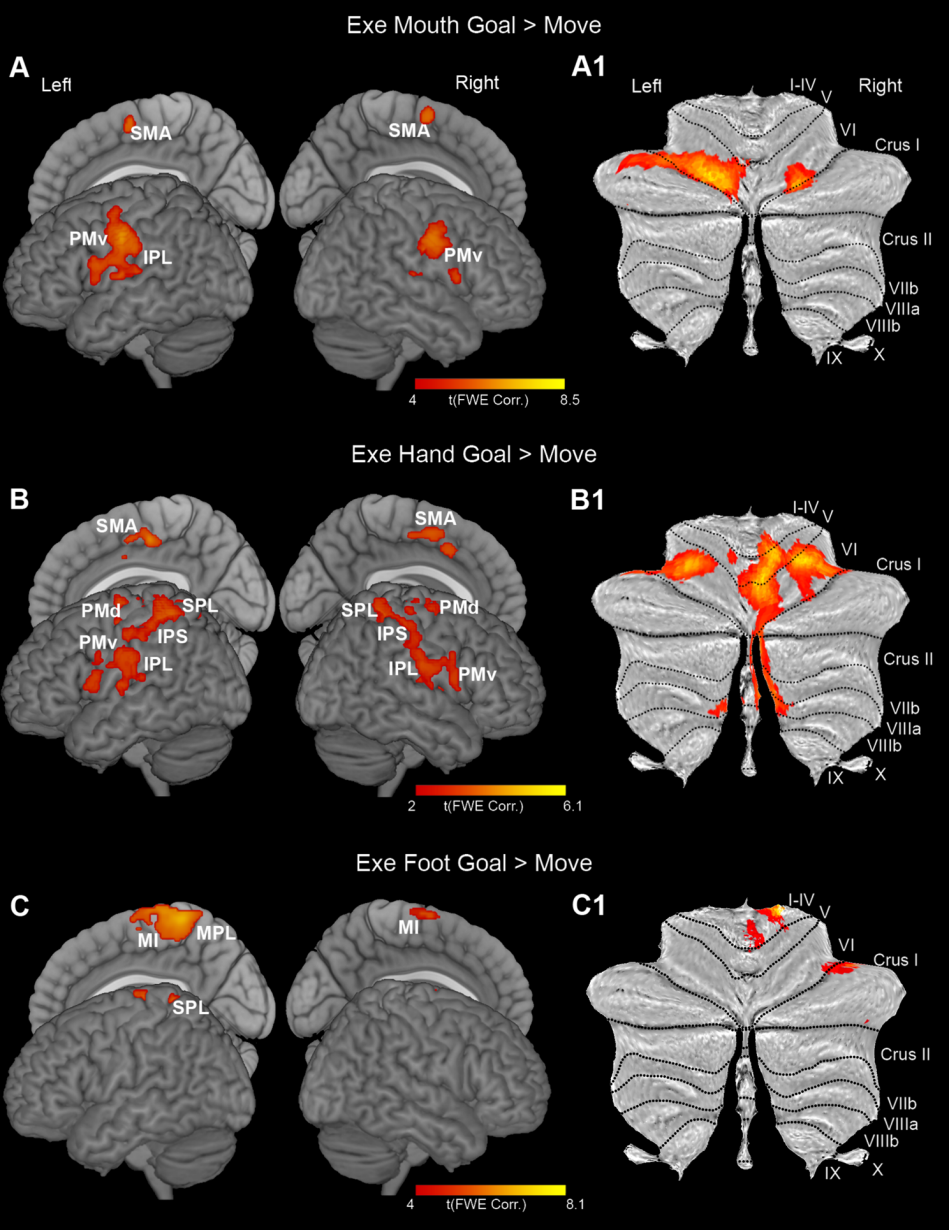
Cortical and cerebellar activations related to the contrasts *Exe Mouth Goal* > *Move* (**A, A1**), *Exe Hand Goal* > *Move* (**B, B1**), *Exe Foot Goal* > *Move* (**C, C1**). Whole-brain statistical parametric maps are rendered on a 3D MNI ch2 brain template (MRIcron software; https://www.nitrc.org/projects/mricron). Cerebellar activations are shown on a flat map of the cerebellum (SUIT, http://www.diedrichsenlab.org). Statistical threshold set at *p* < 0.001 (FWE corrected at cluster level)

In the cerebral cortex, the contrasts *Exe Goal* > *Exe Move* showed activation of motor, somatosensory, and posterior parietal cortices, with a quite clear somatotopic organization in which foot, hand, and mouth are represented in a medio-lateral order (Fig. 2A, B, and C). For each considered effector, the activations were bilateral, although more extended in the left hemisphere, especially for primary sensorimotor areas.

In the cerebellum, the activations observed during the contrast *Exe Goal* vs. *Exe Move* showed a quite clear somatotopic organization (Fig. 2, A1, B1, and C1). Concerning mouth grasping actions, the activated voxels were located bilaterally in the middle part of lobule VI, and, on the left, they extended to the lateral part of this lobule and crus I (Fig. 2, A1) thus involving the anterior sensorimotor representation of the mouth. Concerning hand actions, bilateral activations were found in lobules VI and VIIIa, and, on the right hemisphere, the activated voxels extended to the vermis and intermediate part of lobules I–IV, V, Crus I, Crus II, and VIIb (Fig. 2B1) thus involving the anterior sensorimotor representation of the hand. The local maxima, found in lobules V and VI, were lateralized to the right hemisphere. An additional cluster was found in the right lobule VIIIa, in a territory likely corresponding to the secondary hand motor representation. Concerning foot actions, the activation is very restricted and included a cluster in the right lobules I-IV and another cluster in lobule VI extending laterally to the Crus I (Fig. 2C1).

The direct contrast between couples of effectors during execution of goal-related actions vs. simple movements (Exe Goal vs. Exe Move) showed the presence of significant clusters, in both cerebral cortex and cerebellum, according with the classical somatotopic organization in which foot, hand, and mouth are represented in a medio-lateral order. The results of this analysis are included in the Supplementary Information (Suppl. Figure 10).

**Fig. 3:**
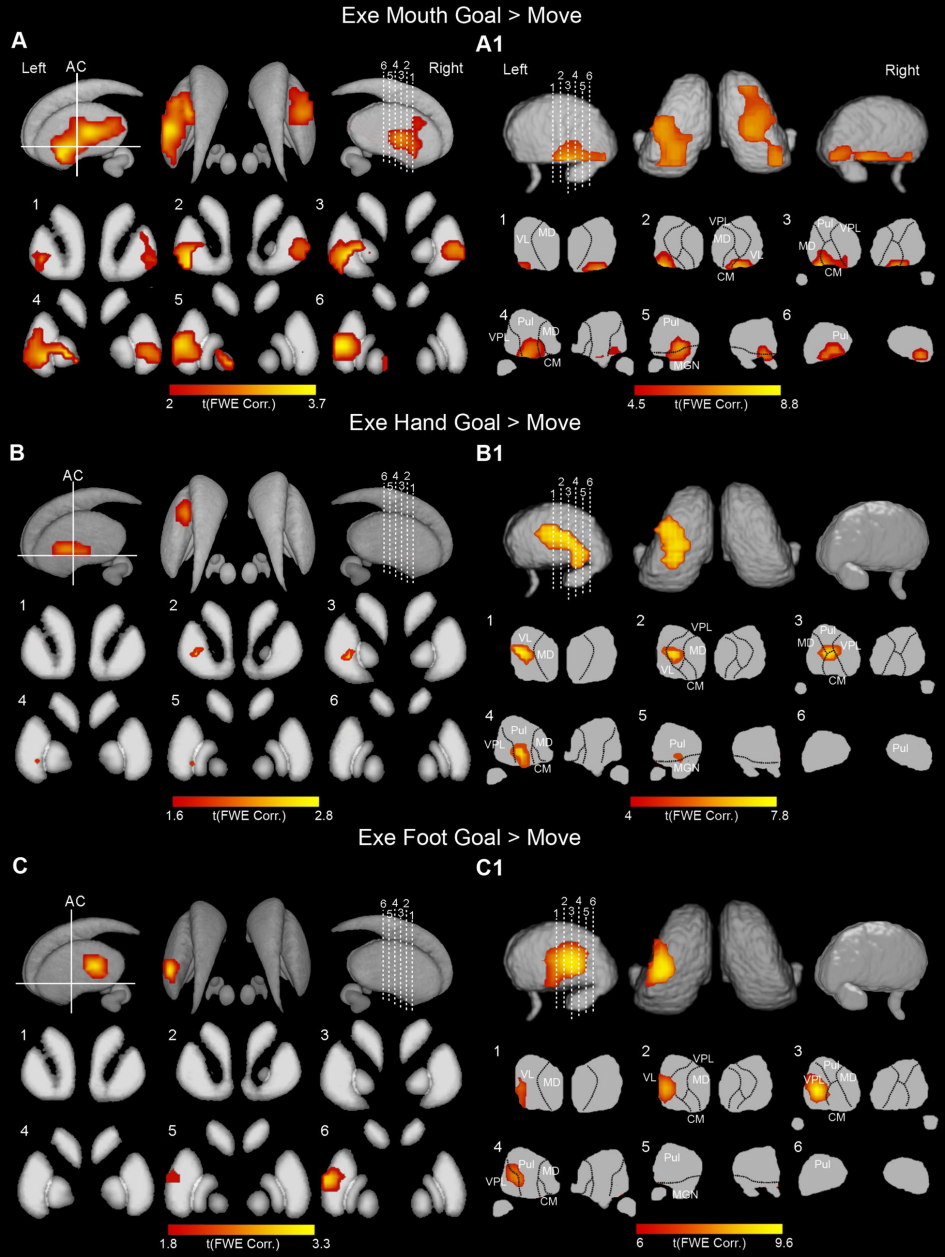
Basal ganglia and thalamic activations related to the contrasts *Exe Mouth Goal* > *Move* (**A, A1**), *Exe Hand Goal* > *Move* (**B, B1**); *Exe Foot Goal* > *Move* (**C, C1**). Basal ganglia activations are shown on a 3D template (Atlas of the basal ganglia, ATAG; https://www.nitrc.org/projects/atag/; left view, right view, and axial view) and six coronal representative sections from ch2 template (MRIcron software; https://www.nitrc.org/projects/mricron). Thalamic activations are shown on a 3D template (Thomas Atlas) (Su et al. 2019), left view, right view, and axial view, and six coronal representative sections from ch2 template. *AC *anterior commissure. Statistical threshold set at *p* < 0.001 (FWE corrected at cluster level)

In the basal ganglia, the activations surviving to the contrast *Exe Goal > Move* involved the putamen and the Globus Pallidus (GP) (Fig. 3A, B, and C). During the execution of mouth grasping actions, the significant clusters involved bilaterally the entire extent of the so-called “motor” putamen, that is the territory located posteriorly to the level of the anterior commissure (AC) and extend to the territory rostral to the AC, especially on the left in which the activations are more extensive (Fig. 3A). In the GP the significant activations were bilateral and involved both the external and the internal parts of the GP (GPe; GPi, respectively). During the execution of hand grasping actions, the activation was limited to the left putamen, where it involved a dorsal and lateral territory, at an antero-posterior level around the location of the AC (Fig. 3B). During execution of foot grasping actions, the activation was completely lateralized to the left putamen, involving exclusively its “motor” territory (Fig. 3C). The local maxima appear to be located slightly more lateral and dorsal than those observed during the execution of hand grasping movements.

In the thalamus, during contrast *Exe Goal > Move* for all effectors, the activations involved the sensorimotor nuclei of this structure (Fig. 3A1, B1, C1), showing a somatotopic organization, with mouth represented more ventrally with respect to the representation of the hand, whose activation, in turn, was located more medial and ventral as compared to that produced by foot movements. Specifically, concerning mouth grasping actions, the activation involved, bilaterally, the ventral part of the ventrolateral nucleus (VL), and the ventral part of the centro-median nucleus (CM) (Fig. 3A1). Concerning hand grasping actions, the activation was found only on the left and involved VL, CM, and the anteriormost part of the pulvinar; the activation in VL and CM extended more dorsally than those observed in mouth condition (Fig. 3B1). During foot grasping actions, the activation was observed only on the left, where it involved the lateral part of VL and ventro-lateral posterior nucleus (VPL; Fig. 3C1).

### Brain Activations During Observation of Grasping Actions

**Fig. 4:**
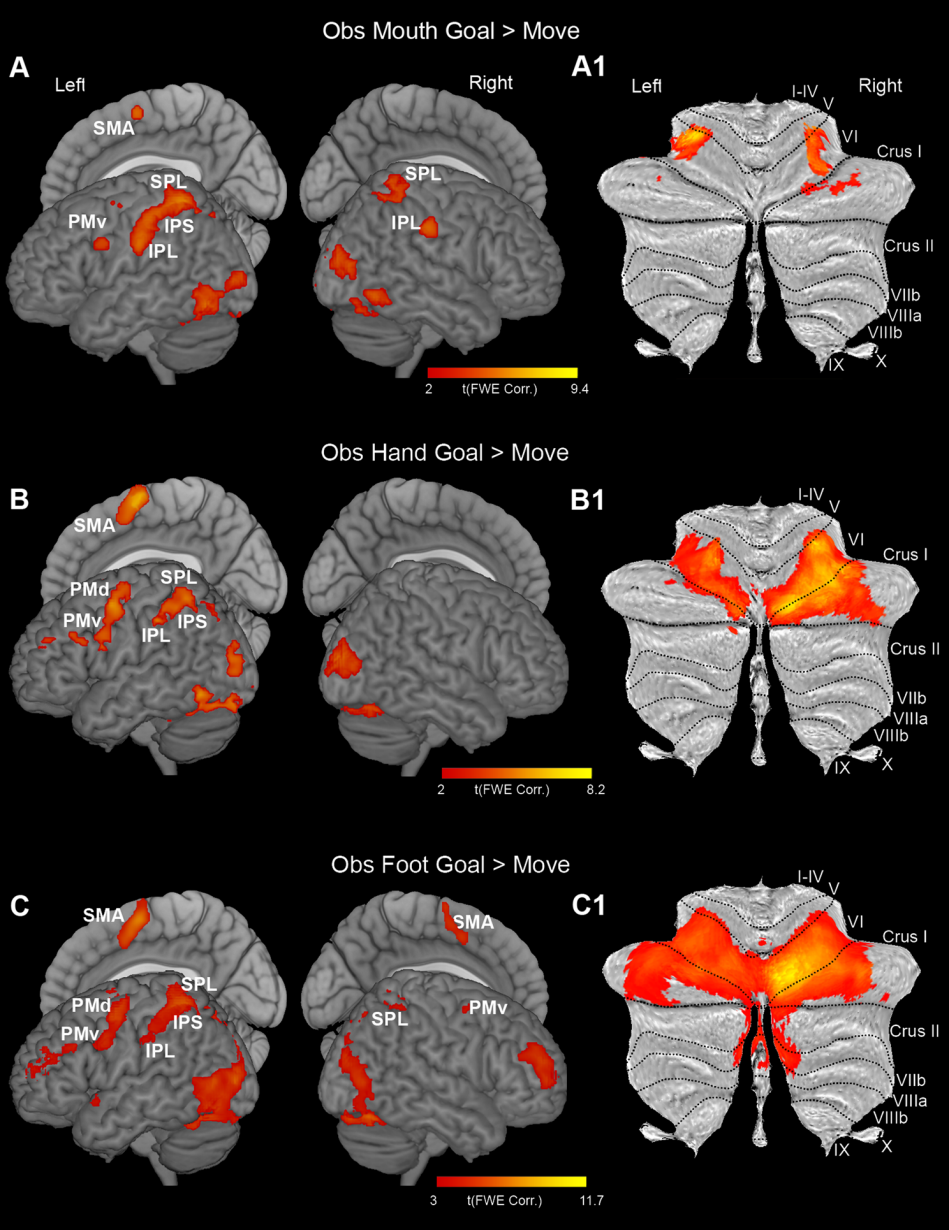
Cortical and cerebellar activations related to the contrasts *Obs Mouth Goal* > *Move* (**A, A1**), *Obs Hand Goal* > *Move* (**B, B1**), *Obs Foot Goal* > *Move* (**C, C1**). Whole-brain statistical parametric maps are rendered on a 3D MNI ch2 brain template (MRIcron software). Cerebellar activations are shown on a flat map of cerebellum (SUIT). Statistical threshold set at *p* < 0.001 (FWE corrected at cluster level)

The cortical activations found during the contrast *Obs Goal > Move* revealed clear parieto-frontal activations in the left hemisphere for all effectors (Fig. 4A, B, and C), with the observation of foot actions also activating a cluster in the dorsolateral prefrontal cortex. In addition, in the right hemisphere, it was evident a strong activation of the posterior parietal cortex in Mouth and Foot conditions and a ventral premotor and prefrontal activation in Foot condition.

The cerebellar activations revealed by the contrasts *Obs Goal > Move* were largely symmetrical in both cerebellar hemispheres, with clusters of activity in lobules V, VI, crus I, crus II, and VIIb (Fig. 4, A1, B1, and C1). In particular, concerning the observation of mouth actions, the activations involved, on both cerebellar hemispheres, the lateral part of lobule VI and a restricted part of adjacent lobule V while, on the right, the lateral part of Crus I (Fig. 4A1). Concerning hand actions, the activated voxels involved bilaterally almost the entire extent of lobule VI, from which they slightly extended to the adjacent lobule V, and, on the right, to the medial and central parts of Crus I (Fig. 4B1). Concerning foot actions, the location of activated voxels is similar to those found following observation of hand action, and, in addition, it slightly extended to medial parts of lobules V, Crus II, and VIIb (Fig. 4C1).

**Fig. 5:**
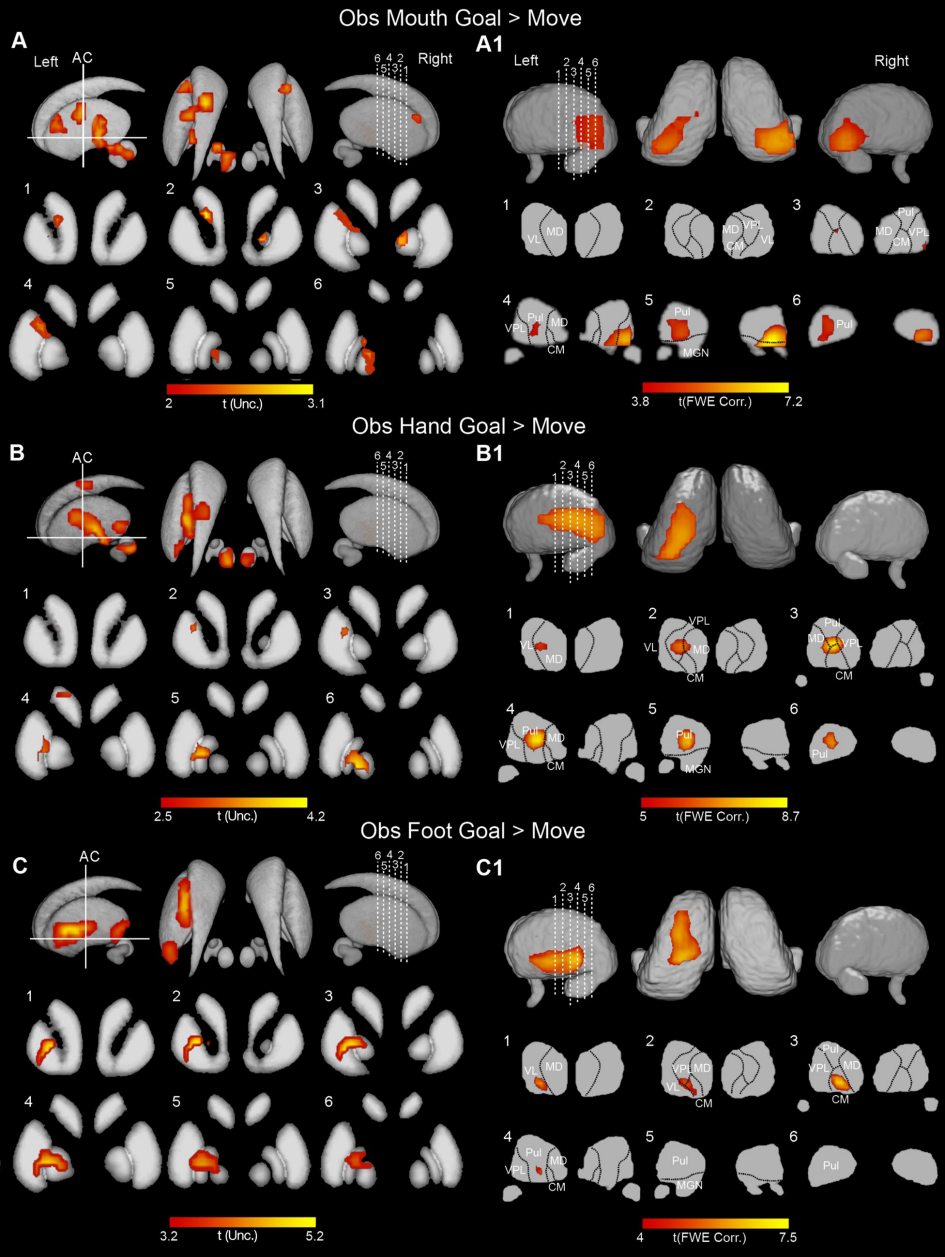
Basal ganglia and thalamic activations related to the contrasts *Obs Mouth Goal* > *Move* (**A, A1**), *Obs Hand Goal* > *Move* (**B, B1**); *Obs Foot Goal* > *Move* (**C, C1**). Basal ganglia activations are shown on a 3D template (ATAG, left view, right view, and axial view) and six coronal representative sections from ch2 template (MRIcron software). Thalamic activations are shown on a 3D template (Thomas Atlas), left view, right view and axial view) and six coronal representative sections from ch2 template. *AC*  anterior commissure. Statistical threshold set at *p* < 0.01 (uncorrected) for basal ganglia activations, and *p* < 0.001 (FWE corrected) for thalamic activations

In the basal ganglia, only using a less restrictive threshold it was possible to find some activations; specifically, in this contrast all activations are rendered with a threshold of *p* < 0.01 (FWE uncorrected for multiple comparisons). Activations were completely lateralized on the left during observation of hand and foot effectors and partially during the observation of mouth movements (Fig. 5, A, B, C). In particular, during observation of goal-related mouth actions, activations involved bilaterally the anterior part of the striatum in a location intermediate between the caudate and the putamen. On the left, additional activations were observed in the motor counterpart of the putamen, in the internal part of GP, and the subthalamic nucleus (Fig. 5A). On the right, in the anterior part of GP. The cluster observed in the motor putamen did not involve the entire medio-lateral sector of this structure, as observed during mouth execution, but is located medially and in the intermediate part of the structure, with respect to the dorso-ventral axis. The activations found during the observation of hand actions involved sectors of putamen and GP quite similar to those found during observation of mouth goal-related actions (Fig. 5B, and C). In addition, the activated pattern also included the external part of GP. Activations during observation of foot actions included different clusters in the putamen, one more posterior and the other rostral to the anterior commissure. Also the external and the internal sector of GP resulted activated.

The thalamic activations found during the contrast *Obs Goal vs. Move* produced an activation pattern bilateral for mouth and restricted to the left for hand and foot. During the observation of mouth action, the activations were almost completely localized within the pulvinar and extended only slightly to adjacent VPL (Fig. 5A1). In hand condition, the activations involved the MD, the VPL, and the pulvinar while in foot condition involved the VL, CM, and pulvinar (Fig. 5B1 and C1).

### Conjunction Analysis Between Observation and Execution

In order to identify the significant voxels showing a shared activation during both execution and observation of goal-related actions vs. simple movements, conjunction analyses (*Exe Goal* > *Exe Move* AND *Obs Goal* > *Obs Move*, for all effectors) were carried out.

**Fig. 6:**
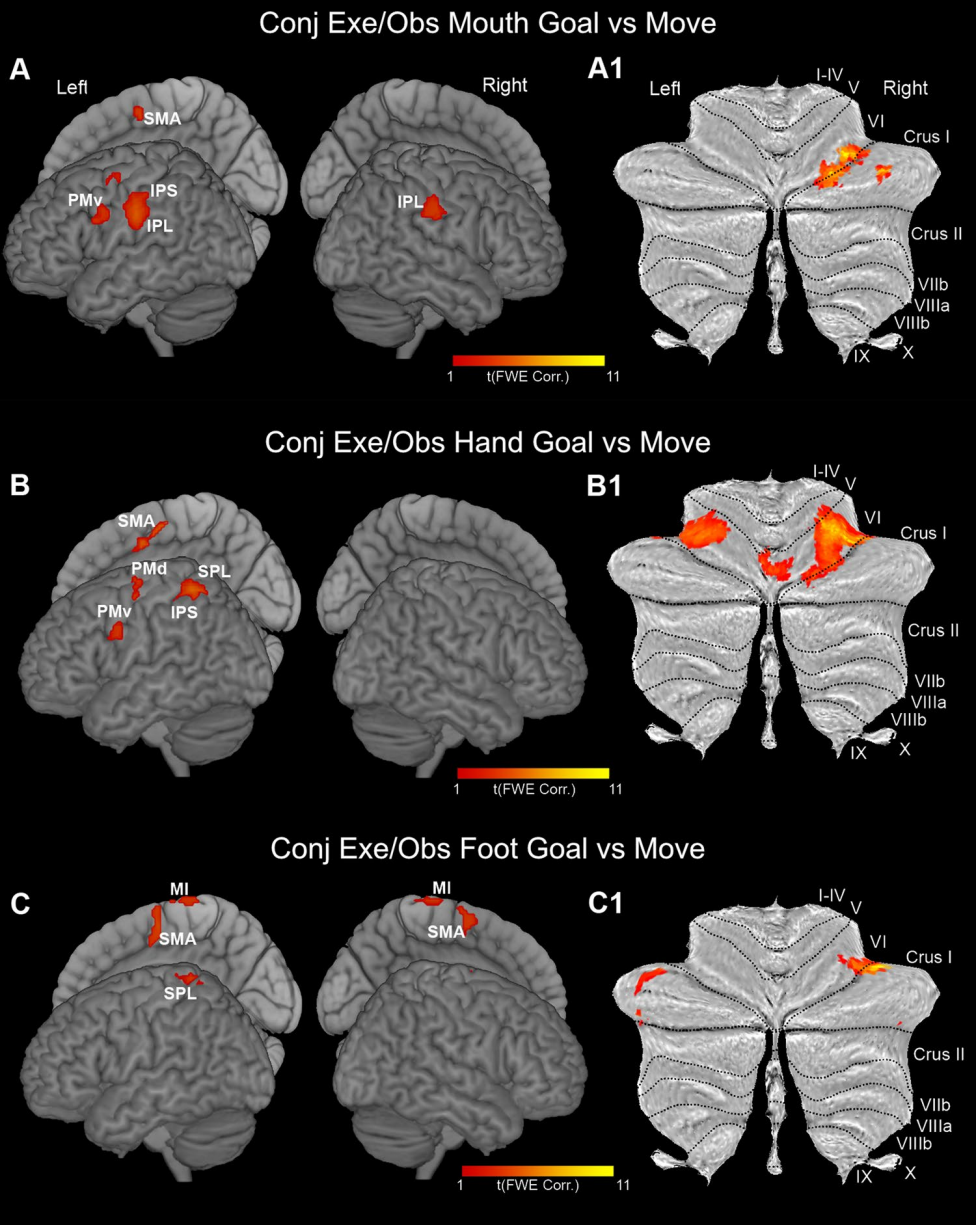
Statistical parametric maps showing the results of the conjunction analysis (cerebral cortex and cerebellum). (**A-A1)** Conjunction map resulted from *Exe Mouth Goal* > *Exe Mouth Move* AND *Obs Mouth Goal* > *Obs Mouth Move*. (**B-B1)** Conjunction map resulted from *Exe Hand Goal* > *Exe Hand Move* AND *Obs Hand Goal* > *Obs Hand Move*. (**C-C1)** Conjunction map resulted from *Exe Foot Goal* > *Exe Foot Move* AND *Obs Foot Goal* > *Obs Foot Move*. Shared activation voxels are rendered on a 3D MNI ch2 brain template (MRIcron software; https://www.nitrc.org/projects/mricron). All activations are rendered with a threshold of *p* < 0.001 (FWE corrected at cluster level)

In the cerebral cortex, the conjunction analysis showed significant shared voxels that differentially involved the nodes of the parieto-frontal MNS, following a rough somatotopic organization in which mouth is represented ventrally and foot dorsally (Fig. 6A, B, C). Concerning the mouth condition, shared significant clusters were observed in PMv, bilaterally, in the left preSMA/SMA, anterior IPS, and IPL (Fig. 6A). The hand condition revealed shared significant clusters in left PMv, PMd, preSMA/SMA, IPL/IPS, and SPL (Fig. 6B). Concerning the foot, shared significant clusters were present bilaterally in the medial part of MI, preSMA/SMA region, and in the left SPL (Fig. 6C). Statistical details and MNI coordinates of significant clusters revealed by conjunction analysis are reported in Supplementary Table 1.

In the cerebellum, the conjunction analysis showed significant shared voxels with partial segregation among the local maxima for different effectors (Fig. 6A1, B1, C1). Concerning the mouth, shared voxels with increased activity were completely lateralized on the right and formed two clusters, one located at the border of lobules VI and Crus I and the other on the central part of Crus I (Fig. 6A1). Concerning the hand, the shared significant clusters were observed bilaterally in the lateral part of lobule VI, from which, on the right, they extended to its ventral and medial parts (Fig. 6B1). Concerning the foot, few shared significant clusters were found, on both cerebellar hemispheres, in the lateral part of Crus I and, on the right, in the lateral part of lobule VI (Fig. 6C1).

**Fig. 7:**
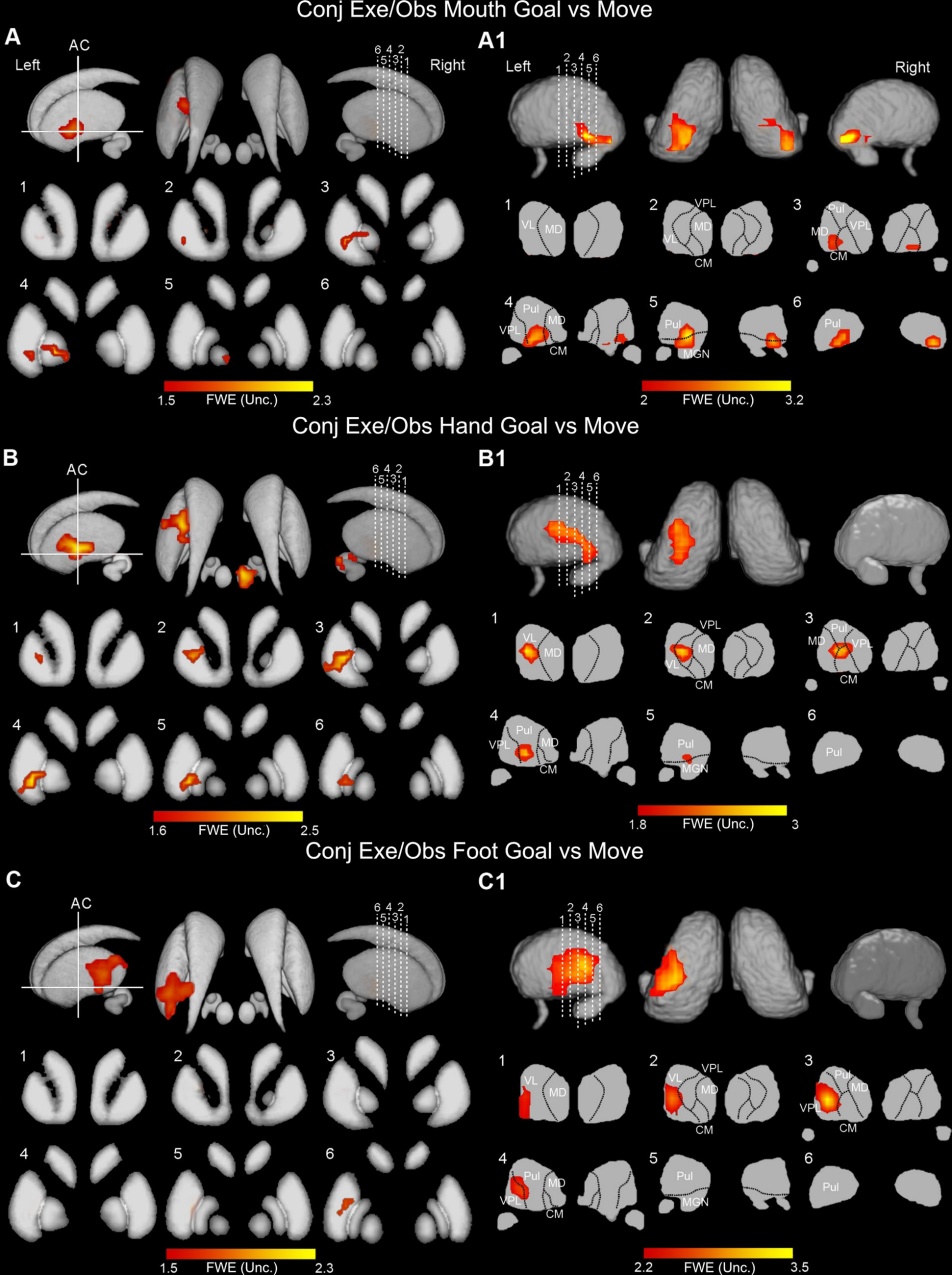
Statistical parametric maps showing the results of the conjunction analysis (basal ganglia and thalamus). (**A-A1)** Conjunction map resulted from *Exe Mouth Goal* > *Exe Mouth Move* AND *Obs Mouth Goal* > *Obs Mouth Move*. (**B-B1)** Conjunction map resulted from *Exe Hand Goal* > *Exe Hand Move* AND *Obs Hand Goal* > *Obs Hand Move*. (**C-C1)** Conjunction map resulted from *Exe Foot Goal* > *Exe Foot Move* AND *Obs Foot Goal* > *Obs Foot Move*. Basal ganglia shared activations are shown on a 3D template (ATAG, left view, right view, and axial view) and six coronal representative sections from ch2 template (MRIcron software). Thalamic shared activations are shown on a 3D template (Thomas Atlas), left view, right view, and axial view) and six coronal representative sections from ch2 template. *AC *anterior commissure. Statistical threshold set at *p* < 0.01 (uncorrected)

In the conjunction analysis carried out on the basal ganglia, only using a less restrictive threshold it was possible to find some activations; specifically, in this contrast all activations are rendered with a threshold of *p* < 0.01 (FWE uncorrected for multiple comparisons). Concerning mouth actions, the activations were lateralized to the left and involved the medial sector of the putamen; additional activations were observed quite extensively in the globus pallidus (Fig. 7A). Concerning hand actions, the activations involved the medial part of the putamen both anteriorly and posteriorly with respect to the AC; further activation was found in the GPe (Fig. 7B). Finally, concerning foot actions, the activations involved almost exclusively the posterior part of the putamen (Fig. 7C).

In the thalamus, the conjunction analysis, using a less restrictive threshold of *p* < 0.01 (FWE uncorrected for multiple comparisons) showed a rough somatotopic organization (Fig. 7A1, B1, and C1). Specifically, in mouth condition shared activated voxels involved almost exclusively the ventral part of the pulvinar and a restricted ventral portion of sensorimotor nuclei, such as VPL, MD, and CM (Fig. 7A1). In hand condition, shared activated voxels were located in the central part of VL, VPL, CM, and anterior pulvinar (Fig. 7 B1). In foot condition shared activated voxels were located in the lateral part of both VL and VPL (Fig. 7C1).

**Fig. 8:**
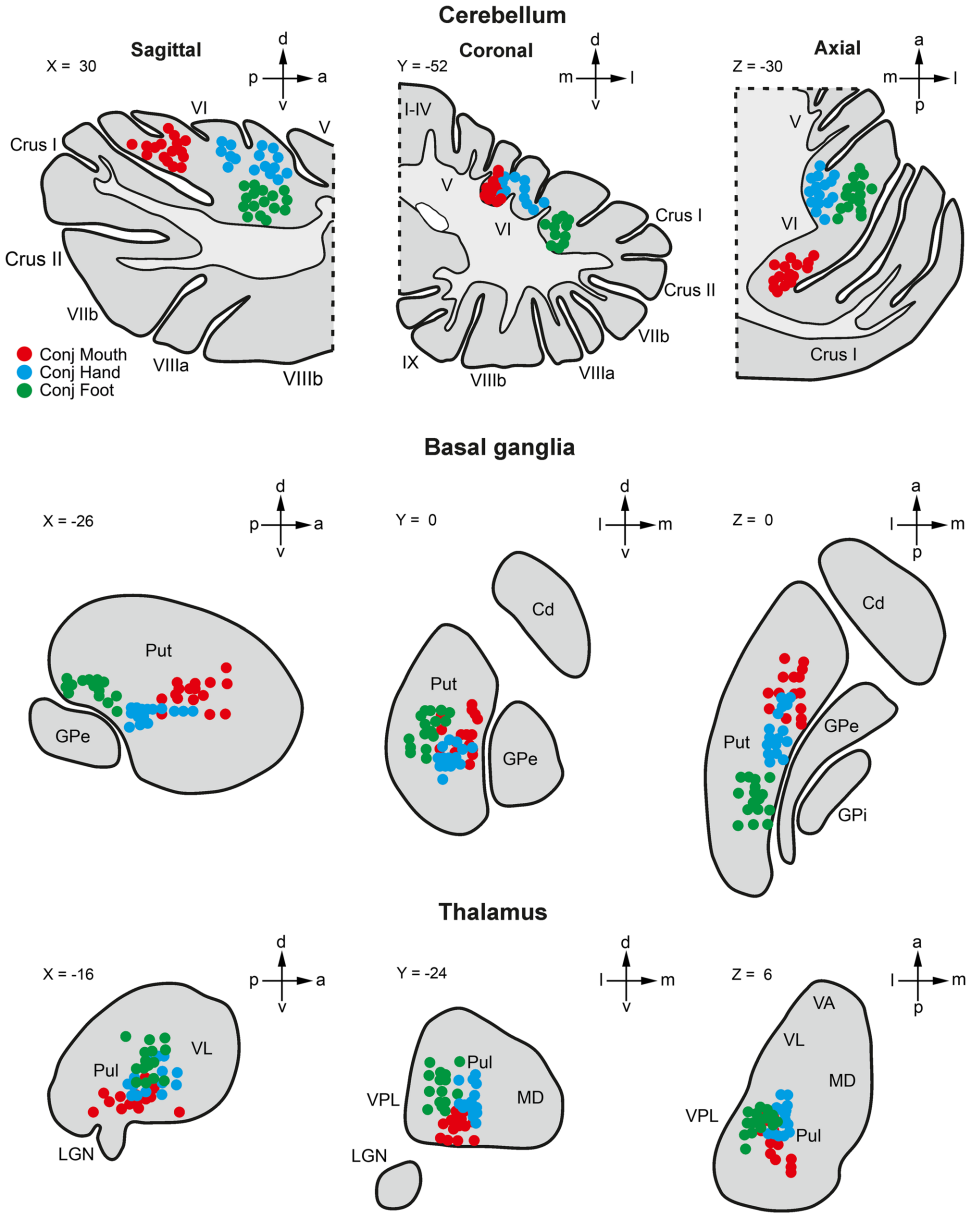
Center of gravity (COG) maps of each participant and effector are reported separately for cerebellum, basal ganglia, and thalamus. The single subject peaks are projected on representative sagittal, coronal, and axial slices

Figure 8 shows a visualization of the CoG of single-subject activation peaks in the cerebellum and subcortical structures related to the conjunction analysis results for Mouth, Hand, and Foot, respectively.

**Fig. 9:**
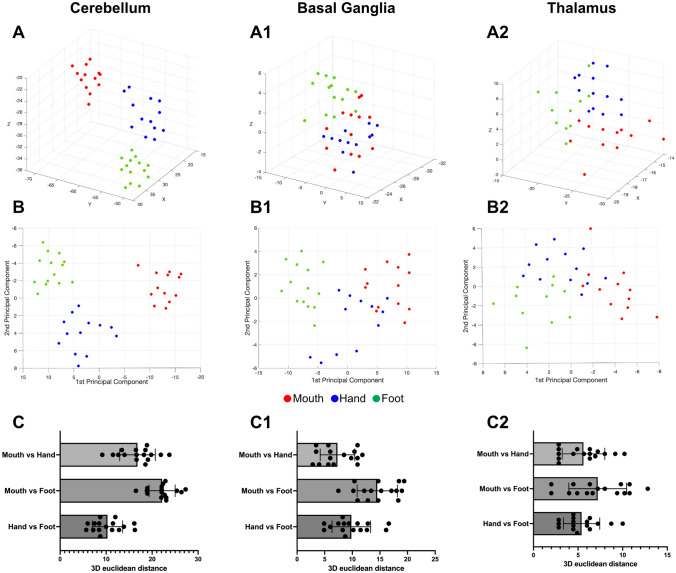
Quantitative measures of the distance between effector representation in the cerebellum and subcortical structures. **(A-A2)** Single subject peak distribution, calculated using conjunction analysis, in 3D space. **(B-B2)** Visual representation of the first and the second principal components projected onto the corresponding plane. **(C-C2)** Euclidean distances between each COG corresponding to the three effectors

In order to further investigate the somatotopic representation of the three effectors within cerebellum, basal ganglia, and thalamus, the single subject peaks were first mapped in the 3D space (Fig. 9A, A1, A2). Then, the single subjects peaks were analyzed with the PCA. The spatial distributions of shared activation peaks between observation and execution along the first and second principal components are shown in Fig. 9B, B1, and B2. Using these transformed coordinates, the differential somatotopic distribution of peak activation in the cerebellum, basal ganglia and thalamus is even more evident. Euclidean distances between the representation of the mouth and the other two effectors in 3D space are shown in Fig. 9C, C1, and C2. The mean distances between activation peaks of the three effectors were significant in all structures, as it follows: cerebellum (Mouth vs. Hand, *t*_(15)_ = 17.4, *p* < 0.001; Mouth vs. Foot, *t*_(15)_ = 31.3, *p* < 0.001; Hand vs. Foot, *t*_(15)_ = 12.4, *p* < 0.001), basal ganglia (Mouth vs. Hand, *t*_(15)_ = 9.3, *p* < 0.001; Mouth vs. Foot, *t*_(15)_ = 15.8, *p* < 0.001; Hand vs. Foot, *t*_(15)_ = 11.2, *p* < 0.001), thalamus (Mouth vs. Hand, *t*_(15)_ = 9.3, *p* < 0.001; Mouth vs. Foot, *t*_(15)_ = 8.8, *p* < 0.001; Hand vs. Foot, *t*_(15)_ = 10.5, *p* < 0.001).

## Discussion

In the present fMRI study, healthy human participants were instructed to execute or to observe grasping acts performed with different effectors, namely mouth, hand, and foot. The main results show that: (1) during execution of goal-related motor acts the activations were somatotopically organized not only at the cortical level but also in the cerebellum, basal ganglia, and thalamus; (2) during action observation no clear cortical, cerebellar or subcortical somatotopic organization was present; (3) when looking for shared activated clusters, a rough somatotopy was found in both cortical, cerebellar and subcortical territories.

### Activations During Action Execution

Cortical activations showed a clear somatotopic organization during execution of grasping motor acts, in line with many previous studies (for a review see (Hardwick et al. 2018)). Similarly, the cerebellar activations during action execution showed, in agreement with the data reported by the literature, a clear somatotopic organization, with the foot located more anterior, the mouth more posterior and medial and the hand more posterior and lateral (Grodd et al. 2001; Schlerf et al. 2010; Mottolese et al. 2013; Guell et al. 2018). The activated territories involved, in the anterior cerebellum, the classical motor representation of each effector and, in the case of the hand, also its secondary motor representation in the posterior cerebellum (Middleton and Strick 1998; Sakai et al. 1998). Furthermore, in the anterior cerebellum, execution of mouth and hand grasping activated lobule VI and Crus I, a territory originally considered exclusively involved in non-motor functions, but recently described as activated also during execution of motor tasks, especially complex ones (Schlerf et al. 2010). The absence of activation of the secondary motor representation is in line with studies reporting that this representation is more involved in motor planning and less activated during movement execution, with respect to the primary one (Stoodley and Schmahmann 2009; Schmahmann et al. 2019).

An unexpected result is the restricted activation observed in the cerebellum during the execution of foot goal-directed movements, indicating that for the foot the cerebellar activation during goal-directed actions and simple movements is comparable. Since also in the cortex the foot activation in the contrast *Goal vs. Move* is quite restricted, a possible explanation is that the movement patterns of the two conditions are quite similar, especially concerning toes. A further possibility is that, in both cortex and cerebellum, the representation of grasping with the foot, being not so much naturalistic, is not as extensive as that of grasping with hand or mouth, that in contrast are used in daily life to take objects and eat food. Whether the use of goal-related actions more appropriate for the foot, such as, for example, pressing a pedal or kicking a ball results in a stronger cerebellar activation remains to be addressed.

Although the cerebellar activations in the contrast *Exe Foot Goal > Move* were quite restricted, they involved unexpectedly also the lateral part of the lobule VI/Crus I, a territory classically not activated during the execution of foot movements (Nitschke et al. 1996; Grodd et al. 2001). A possible interpretation of this activation is the involvement of the toes, differently from previous older studies in which the cerebellar somatotopy was described using foot movements not involving the distal part; in fact, in more recent studies (Guell et al. 2018; Boillat et al. 2020; Saadon-Grosman et al. 2022) in which participants performed toes dorsiflexion and plantar flexion, activation was found in the lateral part of lobule VI in a location similar to that observed here. An additional not mutually exclusive explanation is that in the cerebellum there are more representations of the foot than previously thought.

In the basal ganglia, the activations observed during action execution are quite in line with those observed in the literature (Maillard et al. 2000; Gerardin et al. 2003; Staempfli et al. 2008; Marquis et al. 2019). In particular, in the putamen, the activations showed a quite clear somatotopic organization with the foot located more posterior and lateral, the hand more rostral and medial and the mouth even more rostral and lateral. The mouth activations are bilateral, although stronger on the left, while the hand and foot activations are limited to the left putamen. During the execution of mouth grasping movements, additional activations were observed in the GP. The stronger activation observed in the putamen with respect to GP can be attributed to the capillary circulatory network, that in the putamen is twice as dense as the pallidum (Maillard et al. 2000).

Thalamic activations involved the VL, CM, and VPL nuclei, according to the classical maps of this structure obtained in the monkey using microstimulation approaches (Vitek et al. 1994, 1996). No fMRI study focused on defining, in this structure, the somatotopy of action execution, however, similarly to what was observed by Marquis and colleagues (Marquis et al. 2019), we found that the activations related to mouth motor acts were located more anterior and ventral with respect to the location of those elicited by execution of hand movements, whose activations were, in turn, located more medial and ventral compared to those produced by foot motor acts.

Interestingly, although the territories activated during the execution of mouth actions are bilateral in the cortex as well as in the cerebellum and subcortical structures considered, the observed activations appear stronger and more extensive in the left hemisphere; these results are in line with the classical notion that the left hemisphere is dominant for the voluntary control of facial movements, as those here performed, while the right hemisphere is predominantly activated for spontaneous facial movements triggered by emotions (Gainotti 2019).

### Activations During Action Observation

Our study shows that during action observation the activations of the parieto-frontal MNS nodes are not organized in a clear somatotopic fashion and involve especially the motor hand fields, independently from the observed effector. This result is only in apparent contradiction with a previous study, in which the observation of motor acts performed with different effectors produced clear somatotopic activations (Buccino et al. 2001). In fact, in a more recent experiment it has been shown that when a subject observes a motor act executed with effectors different from those typically used to perform that act, the activations are independent from the observed effector (Jastorff et al. 2010). In other words, when a subject observes a motor act usually performed with the hand, such as grasping, but performed with non-typical effectors, such as the mouth and foot, the cortical activations mainly include areas related to hand motor acts. Thus, the activations appear to reflect the motor meaning and the effector typically associated to that meaning, rather than a mere representation of the observed effector.

Concerning cerebellum, the activations were bilateral for all effectors and wider and stronger especially for hand actions and even more for the foot ones. The activations elicited by the three effectors showed a large overlap, especially in the case of hand and foot. As a whole, the region activated during action observation is located ventrally and laterally with respect to the classical anterior motor representation, extending from the motor representation described in lobule VI and Crus I (Schlerf et al. 2010) to the more cognitive sectors of Crus I (Stoodley and Schmahmann 2009). Our results are in agreement with fMRI studies reporting that the lateral cerebellum is activated during observation of biological motion and manipulative actions, in humans (Vaina et al. 2001; Sokolov et al. 2010, 2012; Abdelgabar et al. 2019; Errante and Fogassi 2020), as well as during observation of grasping actions, in monkeys (Raos and Savaki 2021). Also the lack of activation in the paravermal lobules V/VI and in lobule VIII for action observation here observed, is in line with the aforementioned studies and, as hypothesized by (Raos and Savaki 2021), it may be associated with the metabolic suppression of the forelimb representation in the spinal cord, a phenomenon found exclusively for action observation and not for action execution (Stamos et al. 2010).

The activation of basal ganglia during action observation included the caudate, in the case of mouth and hand actions, as well as the rostral part of motor putamen, in the case of foot actions, the medial sector of the motor putamen and both the external and the internal sectors of GP. In the case of mouth and hand actions, additional activations were observed in the subthalamic nucleus. The activated motor putaminal sector corresponds to the region that in monkey receives projections from PMv and pre-SMA, while the activations found anteriorly to AC and in the caudate are located in the striatal territory that, in monkey, receives projections from the prefrontal cortex (Gerbella et al. 2016; Albertini et al. 2020) and is related to high order functions (Alexander et al. 1986; Tremblay et al. 2015). The activation of internal GP and the subthalamic nucleus, similarly to that already reported in previous works (Errante and Fogassi 2020), indicates that the activation of the indirect pathway in this condition is particularly robust. Since the activation of the indirect pathway plays a crucial role in inhibiting motor acts (Mink 1996), one may speculate that during action observation the GPi activity is involved in decoupling the activations generated in cortical and subcortical motor structures from their outputs, thus, in other words, is devoted to the inhibition of unwanted motor outcomes.

The activations during the observation of foot actions showed a surprising bilateral activation in the prefrontal cortex as well as in the basal ganglia territory recipient from this cortical region and in the cerebellar Crus I, involved in the prefrontal-cerebellar loop. This result agrees with a recent study showing that unexpected stimuli engaged a frontal-basal ganglia network similar to that observed here (Sebastian et al. 2021).

Thalamic activations involved the pulvinar during observation of grasping performed with all the effectors; this nucleus is activated bilaterally for the mouth, while only on the left for the hand and foot. The other nuclei, such as VL, VPL, and MD, were activated on the left during the observation of hand and foot actions but not during the observation of mouth actions. Even for the thalamus, there was no clear evidence of somatotopic segregation. During action observation, the weaker activation of the motor nuclei, compared to that found during action execution, is completely in line with the activation of the indirect and the hyperdirect pathways of basal ganglia, whose increase of activity in fact determines a decrease of the excitatory thalamo-cortical motor signals. In the classical models of the MNS, the visual information reaches its parietal nodes through STS/inferotemporal cortex (Rizzolatti and Luppino 2001; Nelissen et al. 2011). The thalamic activations of the present study suggest that in addition to this cortical pathway, an additional source of visual information to MNS can be represented by the pulvinar. In particular, this subcortical route can represent a fast pathway, independent from and parallel to the cortical temporal streams, allowing to directly convey less detailed visual information to the parietal and premotor MNS nodes.

### Shared Activations During both Action Observation and Execution

**Fig. 10:**
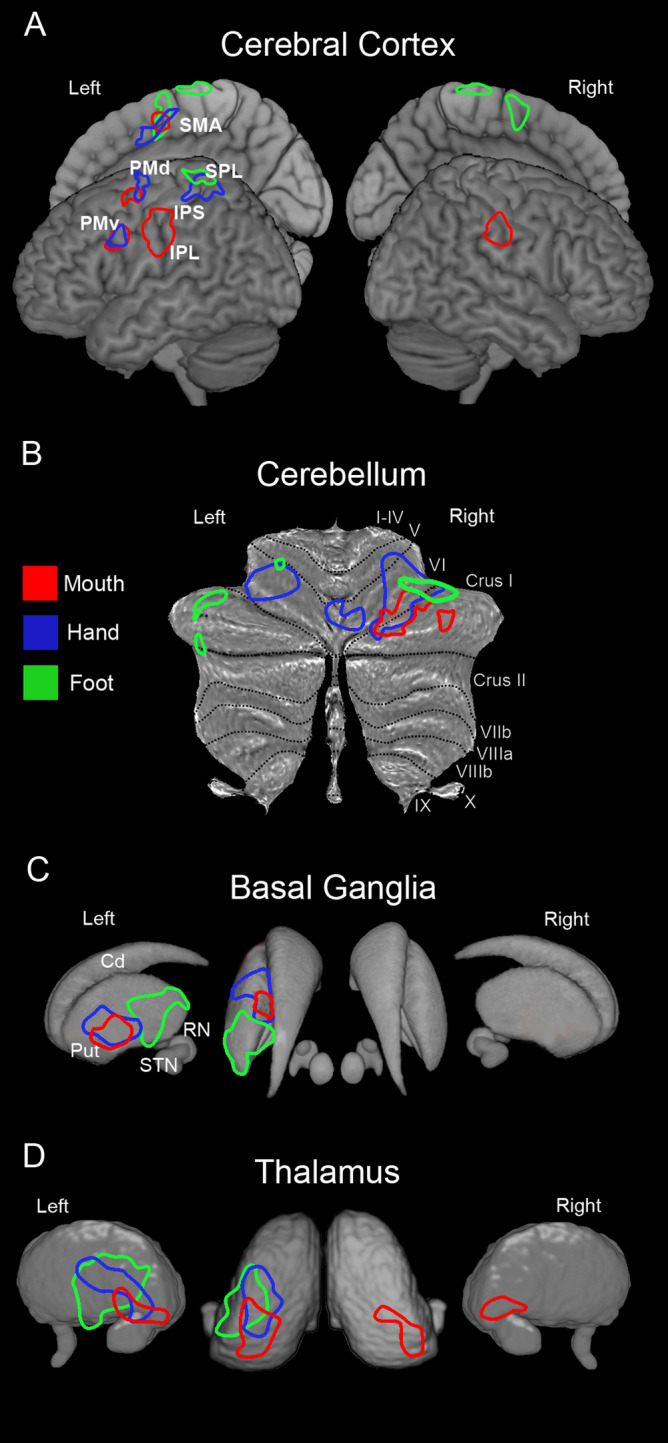
Summary of the conjunction analysis shown in Figs. 6 and 7, related to the shared activations between observation/execution of *goal-related* actions within the cerebral cortex **(A)**, cerebellum **(B)**, basal ganglia **(C)**, and thalamus **(D)**. Contours of clusters related to mouth, hand, and foot activations are shown in red, blue, and green, respectively. *Cd* caudate, *Put* putamen, *RN *red nucleus, *STN* subthalamic nucleus (Color figure online)

A quite evident somatotopic organization in both cortical, cerebellar and subcortical structures has been observed in the territories displaying shared activations during action execution and their observation (Fig. 10). In particular, concerning cortical areas, conjunction analysis revealed: for the mouth, the involvement of the middle part of left PMv, of left preSMA/SMA, and, bilaterally, of the anterior part of IPS/IPL; for the hand the involvement, on the left, of PMd, PMv, preSMA/SMA and the middle part of IPS/IPL; for the foot, the involvement, on the left, of the SPL and bilaterally of the posterior part of the preSMA/SMA and of the adjacent primary sensorimotor sectors (Fig. 10A). Interestingly, the cortical somatotopic organization of the shared activations of mouth and hand resembles that observed in monkey areas containing mirror neurons (Gallese et al. 1996; Ferrari et al. 2003, 2017; Fogassi et al. 2005; Maeda et al. 2015; Papadourakis and Raos 2019).

Shared activations were observed for all three effectors in the preSMA/SMA region. This latter region plays a crucial role in inhibiting or releasing a motor act depending on the context (Rizzolatti et al. 1990; Nachev et al. 2008; Albertini et al. 2020), and is connected with the ventral part of PMv throughout the frontal aslant tract, a bundle known to be involved in the initiation and control of movements (Chernoff et al. 2019; Briggs et al. 2020). An intriguing hypothesis is that these two regions constitute a neural substrate allowing the inhibition or facilitation of actual movements depending on the perceived contextual cues, including observed actions. Although this hypothesis needs to be verified, it could explain why both the pre-SMA/SMA region and the ventral part of PMv are almost always activated during the observation of many types of motor acts independently from the involved effector (Hardwick et al. 2018).

In the cerebellar cortex, the shared activations between observation and execution were similar in both conditions and showed a quite clear somatotopic organization, with the foot located more laterally, the mouth more medially, and the hand in between (Fig. 10B). These shared activations are centered in the lobule VI and Crus I, in the region described as an extent of the classical anterior motor representation and involved in perceiving the kinematics of hand actions performed by others (Schlerf et al. 2010; Abdelgabar et al. 2019; Raos and Savaki 2021). This region, in line with the recent proposal of a modular organization of the primate cerebellar cortex, in which the purely motor areas are located medially and the visuomotor ones laterally (Raos and Savaki 2021), is located between the classical motor cerebellar regions and the sectors in which cognitive functions are represented. This kind of organization appears similar to that observed in the cerebral cortex, in which premotor cortex is located between the primary motor and the prefrontal cortices, suggesting that the above-described cerebellar territory represents a sort of premotor cerebellar region, possibly containing mirror neurons. In line with this hypothesis, this region, although activated during action execution, is not recipient of spinal cord projections, differently from the classical, strictly motor sectors of the anterior cerebellum (Stoodley and Schmahmann 2009). Our data corroborate the findings of a previous study suggesting that a mirror matching mechanism is present also in the cerebellar circuitry, where it might play a role in monitoring the kinematics of an observed action in comparison with that of the same action performed by the observer (Errante and Fogassi 2020). Interestingly, patients with brain lesions involving the lateral part of cerebellum are impaired in tasks requiring the recognition of walking movements presented by point-light displays, in which only kinematics information can be exploited (Sokolov et al. 2010). Accordingly, also patients suffering from spinocerebellar ataxia type 6 were severely impaired in performing tasks in which they had to discriminate subtle differences in the kinematics of observed limb movements (Abdelgabar et al. 2019).

In the basal ganglia, the shared activated regions show a rough somatotopic organization, especially in the putamen, where the mouth is located more anterior, the foot more posterior and the hand in between (Fig. 10C). The shared activated regions in the case of mouth and hand involved territories located around the level of AC, between the classical motor putamen and the part of this nucleus connected to the prefrontal cortex, similarly to what was observed in the cerebellum. This sector is a homolog of that of the monkey putamen receiving projections from all prefrontal, premotor and parietal nodes of the MNS (Gerbella et al. 2016; Bonini 2017). As for the cerebellum, one may speculate that this putaminal territory is a kind of premotor region of the striatum, in line with the notion that in this structure there is a rostro-caudal anatomical organization reflecting that of the cerebral cortex (Draganski et al. 2008; Choi et al. 2012).

In the thalamus, the shared activated region, including the VL, the VPL, the CM nuclei as well as the rostralmost part of the pulvinar, showed a relatively evident somatotopic organization in which the mouth is more ventral, and the hand and the foot more dorsal, with the foot located more laterally than the hand (Fig. 10D). The thalamic nuclei showing mouth and hand shared activations appear to correspond to the thalamic sectors connected, in the monkey, with the mouth and hand mirror neuron networks, respectively (Ferrari et al. 2017; Bruni et al. 2018). The activation of the sensorimotor nuclei (VL, VPL) and of CM, as well as of the rostralmost part of the pulvinar, known to be involved not only in processing visual stimuli but also in the generation of visually guided arm movements (Wilke et al. 2010), suggests that the shared thalamic activation, observed when subjects do not produce any overt or covert movement, can be related to a kind of motor resonance.

Considered together, the shared activated regions in basal ganglia and motor thalamus suggest that observation of already learned actions recruits the same cortico-basal ganglia-thalamo-cortical loop also engaged during execution of the same actions, although during observation overt execution is inhibited.

## Conclusion

Our study confirms preliminary results suggesting that the MNS extends beyond the cerebral cortex to include other regions, such as the cerebellum, basal ganglia, and thalamus, in which it is possible to identify specific, effector-dependent, sectors activated during both action observation and execution, demonstrating that in each of these regions there is a rough somatotopy. On the one side, this would suggest that when the motor system internally simulates an action related to a specific effector, this simulation includes both cortex, cerebellum and subcortical structures. On the other side, it could be suggested that each of these nodes of the MNS can modulate its cortical nodes by processing specific aspects of the observed actions independent of the involved effector, such as monitoring the kinematics of an observed action, in the case of cerebellum, recruiting or inhibiting the overt execution of an observed action, in the case of basal ganglia and sensory-motor thalamus, and/or providing additional, general visual information, in the case of visual thalamus.

A possible limitation of the present study is the use of a standard fMRI protocol to investigate the functional properties of subcortical structures (in particular, basal ganglia and thalamus). In fact, the magnetic properties of these structures are considerably different from cortical regions, suggesting that standard fMRI protocols intended for mapping the cortex are not so optimal to this aim. Future studies, employing combined methods based on submillimeter fMRI and 7T ultra-high field, could deeply investigate the somatotopic organization of subcortex during observation and execution tasks. In addition, adjusted multi-echo fMRI protocols could be used to enhance the accuracy in detecting BOLD activation of iron-rich subcortical regions while maintaining sensitivity within cortical areas (Puckett et al. 2018).

From a translational clinical perspective, these cortico-cerebellar-subcortical mechanisms could be crucial for the recovery of motor functions following brain damage. In the last years, action observation/imitation has been used as a new rehabilitative approach, the so-called Action Observation Treatment (AOT) (Buccino 2014; Buchignani et al. 2019). During a typical AOT session, patients observe a daily life action and afterward reproduce it. So far, this approach has been successfully applied in the rehabilitation of upper limb motor functions in chronic stroke patients, in motor recovery of Parkinson’s disease, and children with cerebral palsy. In principle, AOT has the potential to train actions related to all biological effectors (mouth, upper limbs, lower limbs, and trunk), although so far the focus has been mainly on the recovery of the upper limb. While the role of hand-related cortical areas belonging to the MNS in functional recovery obtained with AOT has been already addressed (Ertelt et al. 2007; Buccino et al. 2018), no studies focused on the possible involvement of cerebellum and subcortical structures in the improvement produced by this protocol. For example, although it has been shown that AOT is effective on many motor deficits of parkinsonian patients, such as bradykinesia, akinesia, and freezing of gait, no information on how the various basal ganglia sectors can affect the motor performance improvement is, up to date, available. To fill this gap, it is needed a detailed description, in healthy subjects, of whether and how cerebellum and subcortical structures are involved during the observation of movements performed using different effectors that are differentially impaired in the various patients and diseases. We hope that our data constitute the first step of it, through the realization of a neurophysiological framework useful not only to understand the possible effect of this protocol in cerebellum and subcortical structures but also to guide subsequent studies addressed to the implementation and refinement of it, based on the types of symptoms and effectors involved in patients.

## Supplementary Information

Below is the link to the electronic supplementary material.

Supplementary file1 (MP4 5082 KB)

Supplementary file2 (MP4 4321 KB)

Supplementary file3 (MP4 3960 KB)

Supplementary file4 (MP4 4005 KB)

Supplementary file5 (MP4 2480 KB)

Supplementary file6 (MP4 2024 KB)

Supplementary file7 (DOCX 8083 KB)

## Data Availability

The data of the present study can be made available, on request, from the corresponding author.
