# Supplementary material for: Activation of Cerebellum, Basal Ganglia and Thalamus During Observation and Execution of Mouth, hand, and foot Actions
Source: Brain Topogr. 2023 May 3;36(4):476–99. doi: 10.1007/s10548-023-00960-1 (PMC10293454; doi:10.1007/s10548-023-00960-1)
Supplement: Supplementary file 7 — Supplementary file7 (DOCX 8083 KB) [file 10548_2023_960_MOESM7_ESM.docx]

Brain Topography

**Activation of cerebellum, basal ganglia and thalamus during observation and execution of mouth, hand, and foot actions**

Antonino Errante^1,2†^, Marzio Gerbella^1†^, Gloria P. Mingolla^3^, Leonardo Fogassi^1*^

*^1^ Department of Medicine and Surgery, University of Parma, Parma, Italy*

*^2^ Department of Diagnostics, Neuroradiology unit, University Hospital of Parma, Parma, Italy*

*^3^ Department of Neurosciences, Biomedicine and Movement Sciences, University of Verona, Verona, Italy*

**Correspondence to:* leonardo.fogassi@unipr.it

**Supplementary Information**

**Supplementary Videos**

**Suppl.Video1_Mouth_Goal.mp4.** Video-clip showing an example of mouth grasping actions performed during the execution task.

**Suppl.Video2_Mouth_Move.mp4.** Video-clip showing an example of mouth simple opening-closing movements performed during the execution task.

**Suppl.Video3_Hand_Goal.mp4.** Video-clip showing an example of hand grasping actions performed during the execution task.

**Suppl.Video4_Hand_Move.mp4.** Video-clip showing an example of hand simple opening-closing movements performed during the execution task.

**Suppl.Video5_Foot_Goal.mp4.** Video-clip showing an example of foot grasping actions performed during the execution task.

**Suppl.Video6_Foot_Move.mp4.** Video-clip showing an example of foot simple opening-closing movements performed during the execution task.

**
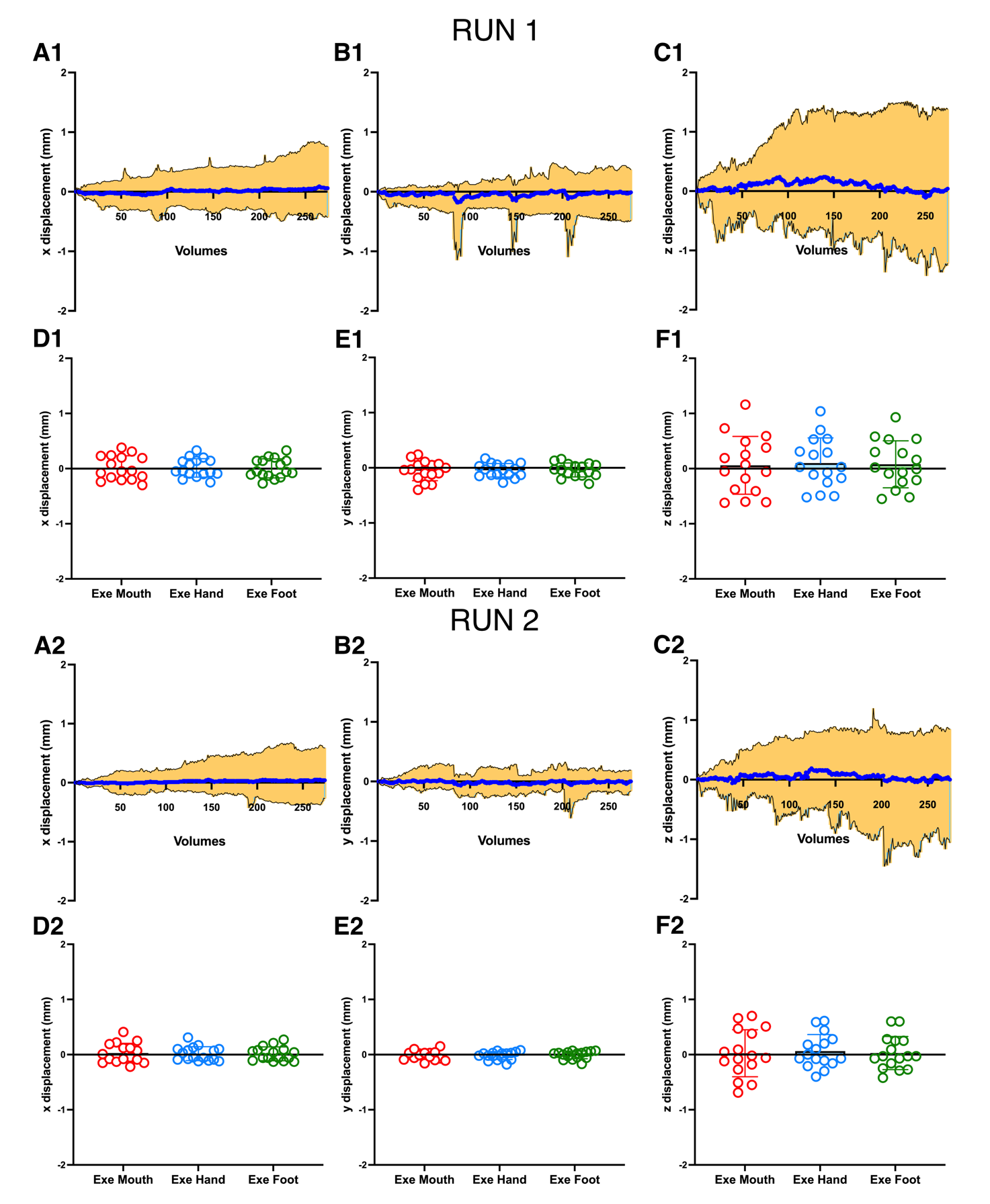
**

**Supplementary Figure 1.** Movement parameters showing head motion during fMRI execution runs. Line graphs show mean movement parameters derived from the realignment step carried out on all subjects separately for Run 1 **(A1, B1, C1)** and Run 2 **(A2, B2, C2)**. Head translation is calculated in mm and represents movement in the x, y and z planes of the scanner. Mean values are depicted as blue line. Orange surface represents the maximum range of movements for all subjects.

Histograms show the mean displacement along the x, y and z planes during the execution of mouth, hand and foot actions, grouped separately for Run 1 **(D1, E1, F1)** and Run 2 **(D2, E2, F2)**. Circles indicate single subject values. Vertical lines correspond to Standard Deviation.

Overall, the head movements during the execution runs were <2mm. The ANOVA shows that no significant differences in amount of movement were present during execution performed with different effectors (RUN1: x displacement, *F*_(2, 45)_=0.85, *P*=0.43; y displacement, *F*_(2, 45)_=0.04, *P*=0.96; z displacement, *F*_(2, 45)_=0.40, *P*=0.66; RUN2: x displacement, *F*_(2, 45)_=1.30, *P*=0.28; y displacement, *F*_(2, 45)_=0.09, *P*=0.90; z displacement, *F(2, 45)*=0.05, *P*=0.94).

**Supplementary Table 1.** Statistical values for GLM group analysis related to cortical activations revealed by conjunction analysis between *Obs Goal vs Move* and *Exe Goal vs Move* conditions. Left column reports the most likely anatomical regions derived from Anatomy Toolbox v.3. Local maxima are given in MNI standard brain coordinates. Significant threshold is set at *p* < 0.001, FWE-corrected for multiple comparisons at cluster-level.

| Anatomical Area | *Left hemisphere* | | | | *Right hemisphere* | | | |
| --- | --- | --- | --- | --- | --- | --- | --- | --- |
|  | z-scores | Peak MNI coordinates | | | z-scores | Peak MNI coordinates | | |
|  |  | x | y | z |  | x | y | z |
| ***Obs Mouth Goal > Move AND***  ***Exe Mouth Goal > Move*** |  |  |  |  |  |  |  |  |
| Inferior Parietal Lobule (PFt) | 5.58 | -62 | -20 | 32 | 3.94 | 58 | -20 | 32 |
| Precentral Gyrus (PMv) | 3.85 | -54 | 4 | 28 |  |  |  |  |
| Supplementary Motor Area | 3.67 | -6 | 2 | 54 |  |  |  |  |
| ***Obs Hand Goal > Move AND***  ***Exe Hand Goal > Move*** |  |  |  |  |  |  |  |  |
| Inferior Parietal Lobule (PFt) | 6.83 | -40 | -32 | 40 |  |  |  |  |
| Intraparietal sulcus (hIP3) |  | -38 | -40 | 44 |  |  |  |  |
| Superior Parietal Lobule (7PC) |  | -36 | -48 | 60 |  |  |  |  |
| Precentral Gyrus (PMd) | 5.36 | -28 | -12 | 64 |  |  |  |  |
| Precentral Gyrus (PMv) | 4.99 | -54 | 4 | 30 |  |  |  |  |
| Pre-supplementary Motor Area | 5.97 | -4 | 4 | 56 |  |  |  |  |
| ***Obs Foot Goal > Move AND***  ***Exe Foot Goal > Move*** |  |  |  |  |  |  |  |  |
| Superior Parietal Lobule (7PC) | 4.91 | -34 | -48 | 62 |  |  |  |  |
| Supplementary Motor Area | 4.37 | -4 | 6 | 54 |  |  |  |  |

**Supplementary Table 2.** Statistical values for GLM group analysis related to activation of subcortical structures revealed by conjunction analysis between *Obs Goal vs Move* and *Exe Goal vs Move* conditions. Left column reports the most likely anatomical regions derived from Anatomy Toolbox v.3. Local maxima are given in MNI standard brain coordinates. Significant threshold is set at *p* < 0.01, uncorrected.

| Anatomical Area | *Left hemisphere* | | | | *Right hemisphere* | | | |
| --- | --- | --- | --- | --- | --- | --- | --- | --- |
|  | z-scores | Peak MNI coordinates | | | z-scores | Peak MNI coordinates | | |
|  |  | x | y | z |  | x | y | z |
| ***Obs Mouth Goal > Move AND***  ***Exe Mouth Goal > Move*** |  |  |  |  |  |  |  |  |
| **Basal Ganglia** |  |  |  |  |  |  |  |  |
| Putamen | 4.44 | -26 | 0 | -4 |  |  |  |  |
| Globus Pallidus |  | -18 | -6 | 0 |  |  |  |  |
| **Thalamus** |  |  |  |  |  |  |  |  |
| VPL/CM | 3.23 | -16 | -26 | 2 |  |  |  |  |
| **Cerebellum** |  |  |  |  |  |  |  |  |
| Lobule VI |  |  |  |  | 3.91 | 30 | -60 | -26 |
| Crus I |  |  |  |  |  | 22 | -74 | -24 |
| ***Obs Hand Goal > Move AND***  ***Exe Hand Goal > Move*** |  |  |  |  |  |  |  |  |
| **Basal Ganglia** |  |  |  |  |  |  |  |  |
| Putamen | 3.67 | -24 | 2 | 0 |  |  |  |  |
| Globus Pallidus |  | -18 | -6 | -2 |  |  |  |  |
| **Thalamus** |  |  |  |  |  |  |  |  |
| Pul/VPL/CM | 2.98 | -14 | -22 | 6 |  |  |  |  |
| **Cerebellum** |  |  |  |  |  |  |  |  |
| Lobule VI | 5.27 | -26 | -52 | -28 | 6.69 | 28 | -50 | -26 |
| Crus I |  |  |  |  |  | 26 | -66 | -28 |
| ***Obs Foot Goal > Move AND***  ***Exe Foot Goal > Move*** |  |  |  |  |  |  |  |  |
| **Basal Ganglia** |  |  |  |  |  |  |  |  |
| Putamen | 3.24 | -28 | -8 | 4 |  |  |  |  |
| Globus Pallidus |  | -20 | 0 | 2 |  |  |  |  |
| **Thalamus** |  |  |  |  |  |  |  |  |
| VPL/VL | 3.51 | -18 | -24 | 8 |  |  |  |  |
| **Cerebellum** |  |  |  |  |  |  |  |  |
| Lobule VI |  |  |  |  | 3.60 | 32 | -52 | -34 |

**Cortical activations vs rest**

During execution conditions, the contrasts *Exe Goal* > *Rest* and *Exe Move* > *Rest* showed activations of motor, somatosensory, and posterior parietal cortices, with a clear somatotopic organization in which foot, hand, and mouth are represented in a medio-lateral order (Suppl. Fig. 2). In particular, Hand and Foot conditions evoked activations more lateralized to the left hemisphere, while Mouth actions and movements elicited more bilateral activations (Suppl. Fig. 2 A, B, C, A1, B1, and C1).


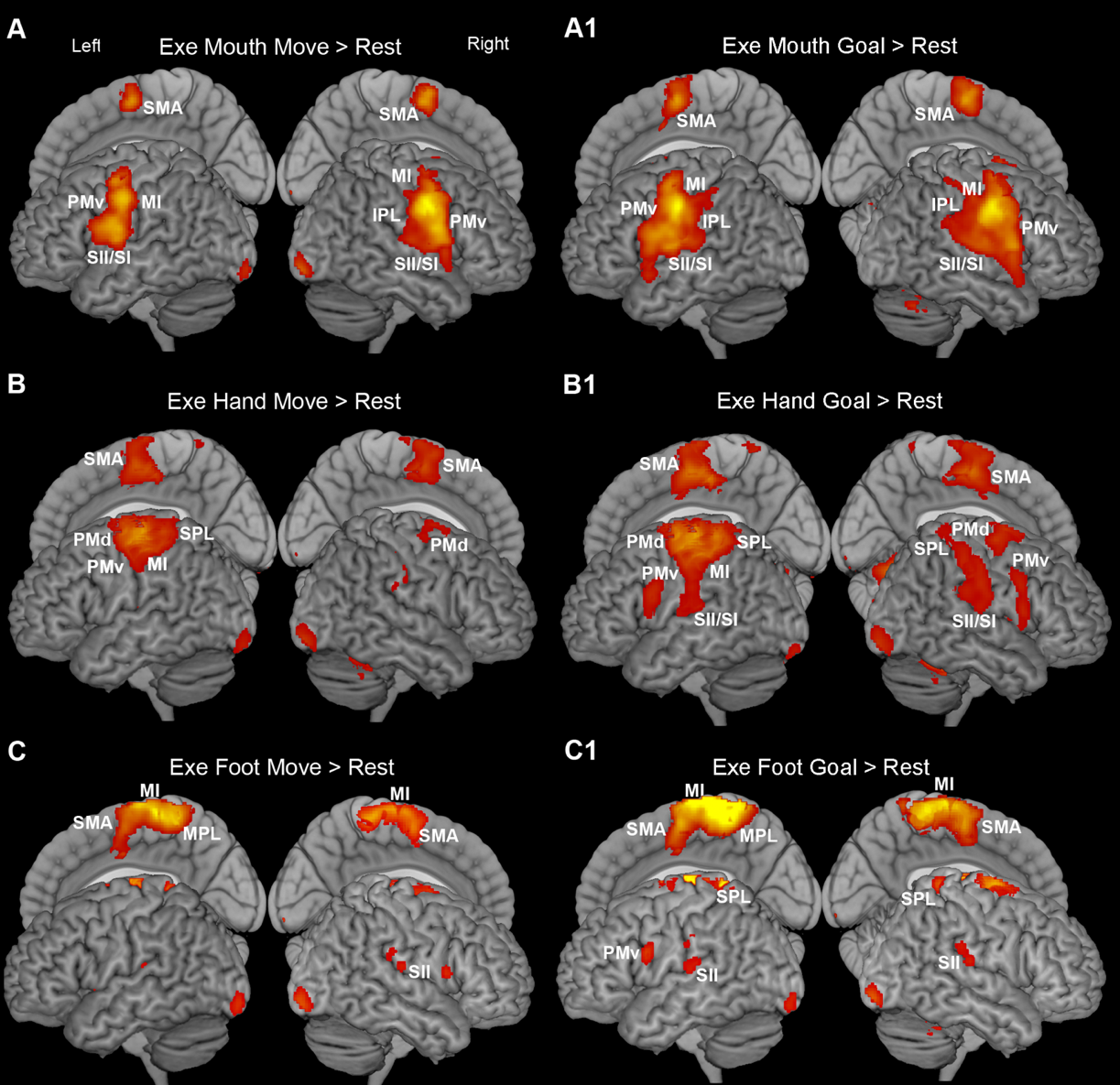


**Supplementary Figure 2.** Cortical activations related to the contrasts *Exe Mouth Goal* > *Rest* (**A**), *Exe Mouth Move* > *Rest* (**A1**), *Exe Hand Goal* > *Rest* (**B**); *Exe Hand Move* > *Rest* (**B1**); *Exe Foot Goal* > *Rest* (**C**) and *Exe Foot Move* > *Rest* (**C1**). Activations rendered on 3D MNI ch2 brain template (MRIcron software; https://www.nitrc.org/projects/mricron). Statistical threshold set at *p*<0.001 (FWE corrected at cluster level).

During observation conditions, the cortical activations found during the contrast *Obs* > *Rest* are quite different between the goal-related and simple movements conditions, with the former producing stronger and more extensive activations than the latter (Suppl. Fig. 3). In particular, during the observation of all types of *goal-related* actions significant voxels have been found, besides visual cortex, in the main nodes of the Mirror Neuron System (MNS), without evidence of any somatotopic organization (Suppl. Fig. 3 A, B, and C). During observation of all types of *simple movements* significant voxels involved, beyond the visual cortex, only partially the nodes of the parieto-frontal MNS (Suppl. Fig. 3 A1, B1, and C1).


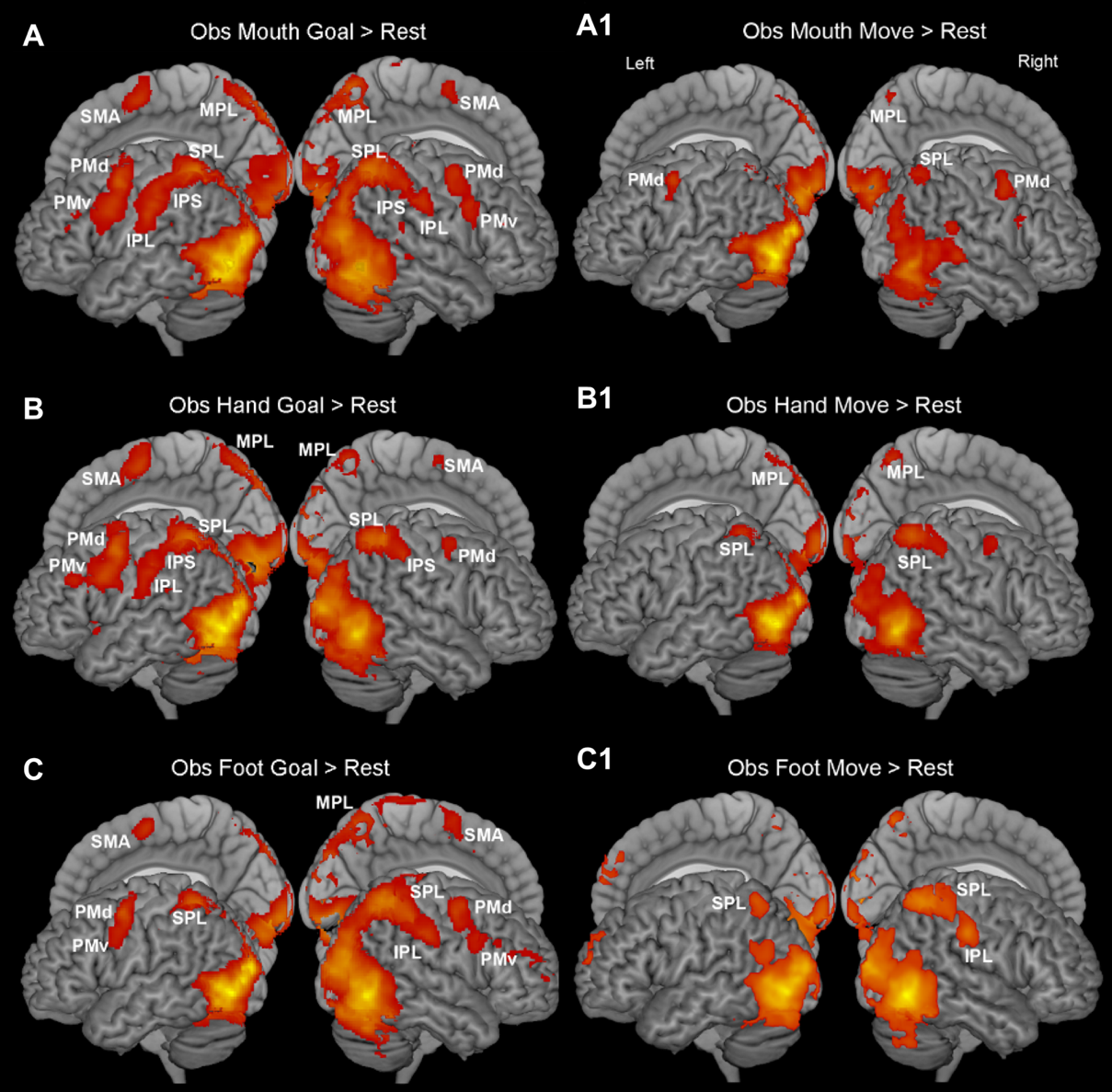


**Supplementary Figure 3.** Cortical activations related to the contrasts *Obs* *Mouth Goal* > *Rest* (**A**), *Obs* *Mouth Move* > *Rest* (**A1**), *Obs* *Hand Goal* > *Rest* (**B**); *Obs* *Hand Move* > *Rest* (**B1**); *Obs* *Foot Goal* > *Rest* (**C**) and *Obs* *Foot Move* > *Rest* (**C1**). All activations are rendered with a threshold of *p*<0.001 (FWE corrected at cluster level).

**Cerebellar activations vs rest**

In the cerebellum, the activations observed during the contrast *Exe* > *Rest* in the *goal-related* conditions for the three effectors are very similar to those observed during the *simple movements* conditions (Suppl. Fig. 4). During execution of mouth actions in both *goal-related* and *simple movements* conditions, the activation pattern was largely symmetrical in both cerebellar hemispheres, with a cluster of activity in lobule VI extending to lobule V and crus I, thus involving the anterior sensorimotor representation of the mouth; the local maxima are lateralized to the left hemisphere and localized in lobule VI (Suppl. Figure 4 A, A1). During execution of hand actions, the activation pattern included a large bilateral cluster in lobules IV, V, VI, thus involving the anterior sensorimotor representation of the hand; the local maxima, found in lobules V and VI, were lateralized to the right hemisphere. Additional clusters were found in right lobules VIIIa and VIIIb, in a territory likely corresponding to the secondary hand motor representation (Suppl. Fig. 4 B, B1). During execution of foot actions, the activation pattern was quite symmetrical in both cerebellar hemispheres, with clusters of activity found in lobules I-IV, V, VI, and crus I; the local maxima, found in lobules I-IV and VI, were lateralized to the right hemisphere (Suppl. Fig. 4 C, C1).

**
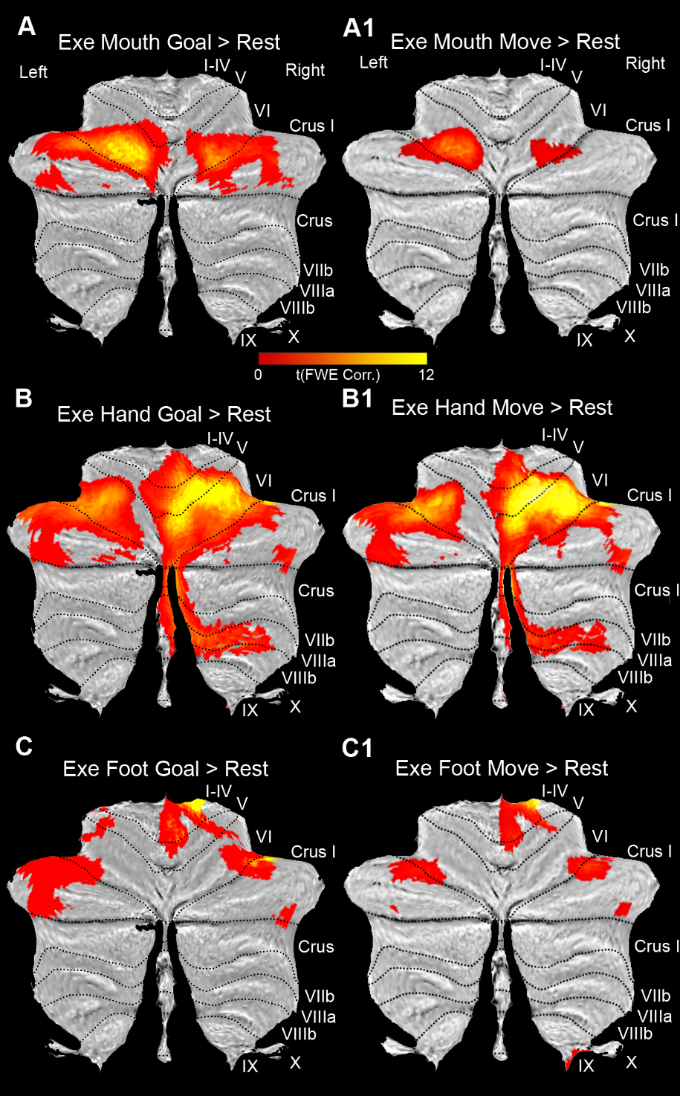
**

**Supplementary Figure 4.** Cerebellar activations related to the contrasts *Exe Mouth Goal* > *Rest* (**A**), *Exe* *Mouth Move* > *Rest* (**A1**), *Exe Hand Goal* > *Rest* (**B**); *Exe Hand Move* > *Rest* (**B1**); *Exe Foot Goal* > *Rest* (**C**) and *Exe Foot Move* > *Rest* (**C1**). Activations are shown on a flat map of cerebellum (SUIT, http://www.diedrichsenlab.org/imaging/suit.htm). Statistical threshold set at *p*<0.001 (FWE corrected at cluster level).

The cerebellar activations found during the contrast *Obs* > *Rest* in the *goal-related* conditions were quite similar for all observed effectors. The activation pattern was largely symmetrical in both cerebellar hemispheres, with clusters of activity in lobules V, VI, crus I, crus II and VIIb (Suppl. Figure 5, A, B and C). The local maxima are bilateral and located in lobule VI and Crus I. Concerning the *simple movements* conditions, the main difference with respect to *goal-related* conditions is that the activated clusters found during observation of the former appeared to be less extensive than those found during observation of the latter (Suppl. Fig. 5, A1, B1 and C1). In particular, concerning mouth and hand, the left activated voxels had a location very similar to that observed in *goal-related* conditions while, on the right, the activated clusters were almost exclusively located in lobules VI and Crus I (Suppl. Figure 5, A, B, A1 and B1). Concerning foot condition, in both cerebellar hemispheres the activated voxels were almost exclusively located in lobules VI, Crus I, and Crus II (Suppl. Fig. 5, C and C1).

**
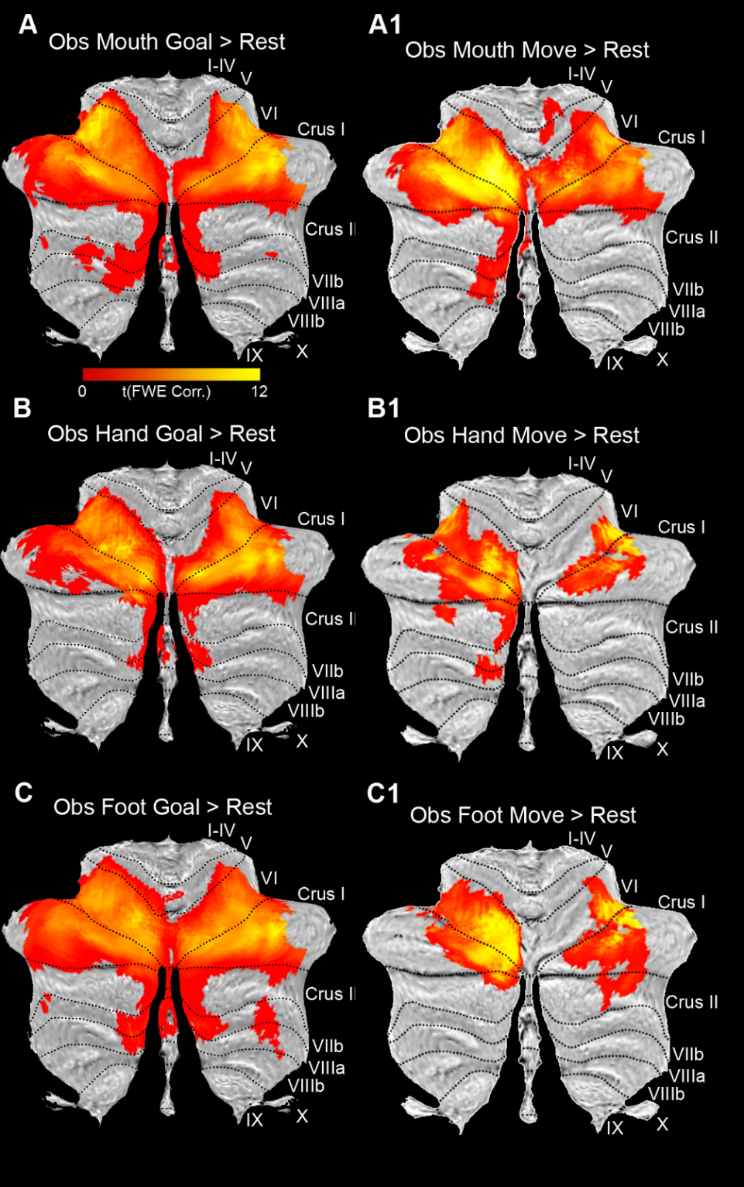
**

**Supplementary Figure 5.** Cerebellar activations related to the contrasts *Obs Mouth Goal* > *Rest* (**A**), *Obs Mouth Move* > *Rest* (**A1**), *Obs Hand Goal* > *Rest* (**B**); *Obs Hand Move* > *Rest* (**B1**); *Obs Foot Goal* > *Rest* (**C**) and *Obs Foot Move* > *Rest* (**C1**). Statistical threshold set at *p*<0.001 (FWE corrected at cluster level).

**Basal ganglia activations vs rest**

In the basal ganglia, the activations observed during contrast *Exe* > *Rest*, in both *goal-related* and *simple movements* conditions for all actions performed, involved the putamen and the Globus Pallidus (GP) (Suppl. Fig. 6).

During the execution of mouth *simple movements*, the significant clusters involved bilaterally the entire extent of the so-called “motor” putamen, that is the territory located posteriorly to the level of anterior commissure (AC), and, on the left, also the territory rostral to the AC (Suppl. Fig. 6 A1). Although the activations were bilateral, the local maxima were lateralized to the right. In motor putamen, the activations become more medial and ventral when moving caudally. In the GP the significant activations are bilateral in in its external segment (GPe) and, on the right, partially extended to its internal segment (GPi) (Suppl. Fig. 6 A1). The putamen and GP activations observed during the execution of *goal-related* mouth actions are similar but more extensive than those observed during the execution of *simple movements* (Suppl. Fig 6 A). In particular, not only the motor putamen but also the sector anterior to AC was activated bilaterally, although in this latter sector the right cluster extended more rostrally as compared to the left cluster. Similarly, to that observed in *simple movements* condition, also during the goal-related one, the activations tended to be more medial and ventral when moving caudally. The local maxima in goal-related condition were bilateral. During the execution of goal-related actions the significant activations were bilateral also in GP, where they involved mainly GPe and marginally GPi (Suppl. Figure 6 A).

During the execution of hand *simple movements*, significant clusters in putamen were completely lateralized to the left, involving the entire extent of the so-called “motor” putamen and extending into the territory rostral to the AC for about 6 mm (Suppl. Fig. 6 B1). The local maxima were located slightly more lateral than those observed during the execution of mouth *simple movements*. Significant activations extended also to GPe mainly in its caudal part (Figure 6 B1). During execution of goal-related hand actions, the significant cluster in left putamen is similar, but more extended, both in caudal and rostral direction, than that observed during the execution of *simple movements* (Figure 6 B). Furthermore, an additional cluster was observed in right putamen involving the sector rostral to AC for about 9 mm. The local maxima were lateralized to left putamen. The significant activations in left GPe are similar to those observed during the execution of *simple movements*, thus involving more extensively the caudal part of the structure; in this condition there was also a relatively small activation of the rostral right GPe (Figure 6 B). The local maxima in GPe were completely lateralized to the left.

During execution of foot *simple movements* activations were completely lateralized to left putamen, involving both its “motor” territory and, for about 6 mm in antero-posterior direction, the territory rostral to AC (Figure 6 C1). The local maxima were located slightly more lateral and dorsal than those observed during the execution of hand *simple movements*. Significant activations in GP involved a very small caudal territory between GPe and GPi, on the left (Figure 6 C1). During execution of goal-related foot actions, the activation was very similar to that observed during the execution of foot *simple movements* (Figure 6 C). In fact, it was observed the same left lateralization as well as a similar medio-dorsal location and a comparable antero-posterior extent. In GP, the significant activation was very small and involved only left GPi (Figure 6 C).


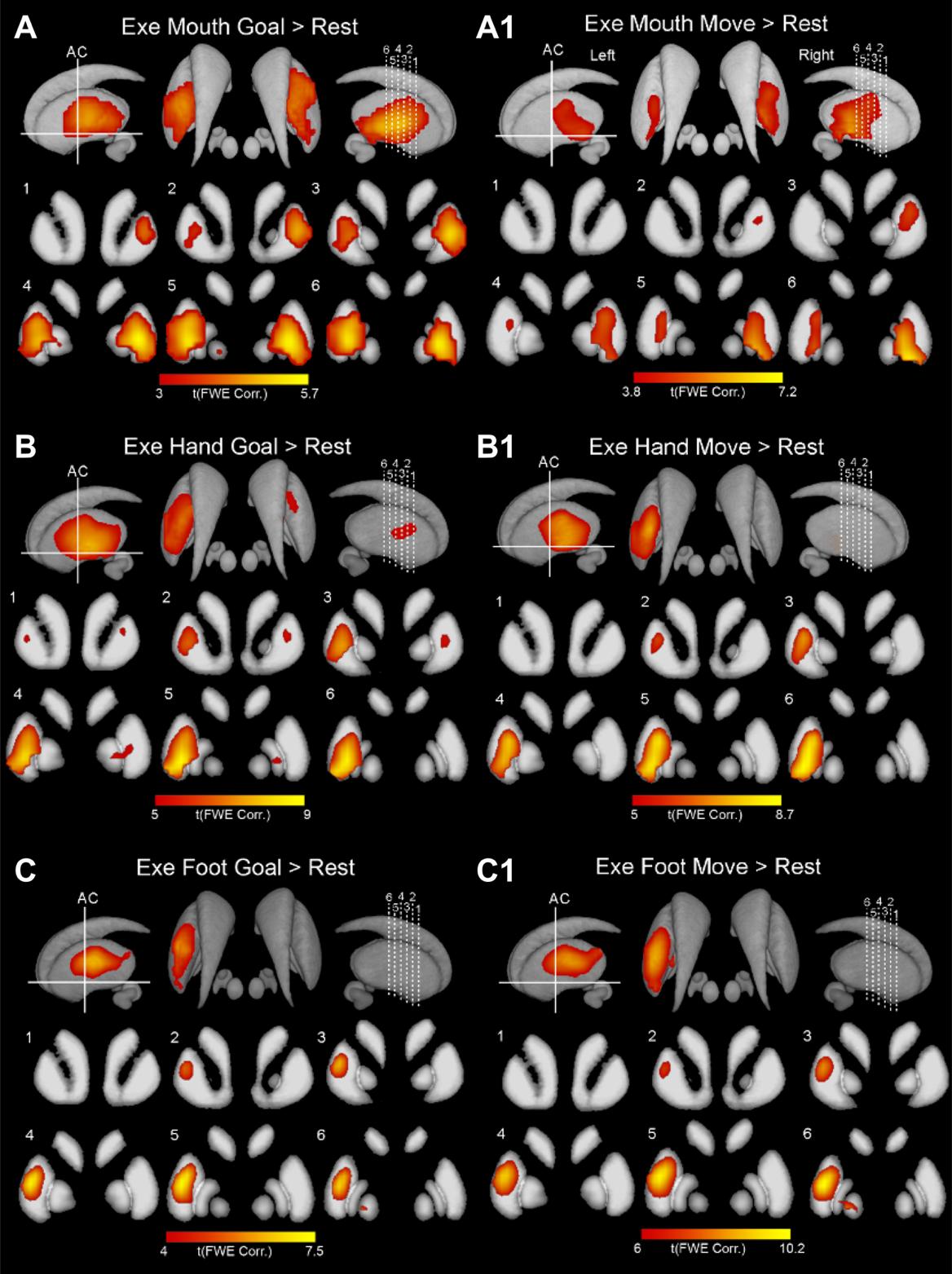


**Supplementary Figure 6.** Basal Ganglia activations related to the contrasts *Exe Mouth Goal* > *Rest* (**A**), *Exe Mouth* *Move* > *Rest* (**A1**), *Exe Hand Goal* > *Rest* (**B**); *Exe Hand Move* > *Rest* (**B1**); *Exe Foot Goal* > *Rest* (**C**) and *Exe Foot Move* > *Rest* (**C1**). Basal ganglia activations are shown on 3D template (Atlas of the basal ganglia, ATAG; https://www.nitrc.org/projects/atag/; left view, right view, and axial view) and six coronal representative sections from ch2 template (MRIcron software; https://www.nitrc.org/projects/mricron). AC = anterior commissure. Statistical threshold set at *p*<0.001 (FWE corrected at cluster level).

During the contrast *Obs* > *Rest* basal ganglia are activated only in *goal-related* condition (Figure 7). During observation of *goal-related* mouth actions, significant activations were bilaterally found in putamen, in caudate and in both GPe and GPi (Figure 7 A). The cluster in putamen only partially corresponded to that observed during the execution of goal-related mouth actions, in fact, it involved only the rostralmost part of the “motor” putamen, and extended to almost the entire territory rostral to AC. Furthermore, the observed cluster did not involve the entire medio-lateral sector of the putamen, as observed during mouth execution, but is located medially and in the intermediate part of the structure, with respect to the dorso-ventral axis (Figure 7 A). The caudate cluster involved the rostralmost part of the structure, in an intermediate position along the dorso-ventral axis and near the internal capsule. The cluster in GP extended along its entire extent, involving both GPe, mainly in the dorsal part, and GPi (Figure 7 A).

The significant activations found during the observation of hand actions involved sectors of putamen and GP similar to those found during observation of mouth goal-related actions, although completely lateralized to the left (Figure 7 B). Furthermore, in contrast to that found after the observation of mouth actions, no significant activation was present in the caudate.

The significant activations found during the observation of foot actions were very similar to those observed during the observation of hand actions, involving analogues sectors of left putamen and left GP and being absent in the caudate (Figure 7 C).


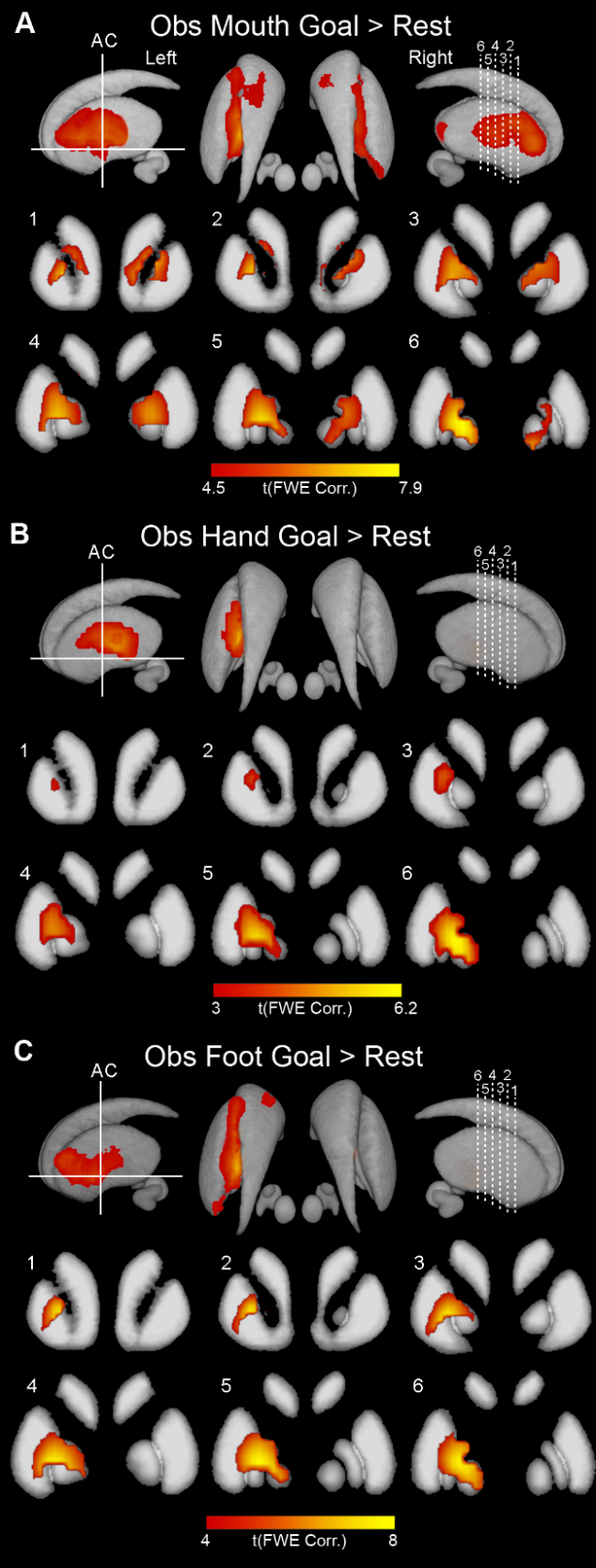


**Supplementary Figure 7.** Basal Ganglia activations related to the contrasts *Obs Mouth Goal* > *Rest* (**A**), *Obs Hand Goal* > *Rest* (**B**), and *Obs Foot Goal* > *Rest* (**C**). All activations are rendered with a threshold of *p*< 0.001 (FWE corrected at cluster level).

**Thalamus activations vs rest**

In the thalamus, during contrast *Exe* > *Rest*, for all actions performed*,* the activations involved the sensorimotor nuclei of this structure (Suppl. Fig. 8), showing a somatotopic organization, with mouth represented more rostrally and ventrally with respect to the representation of the hand, whose activations, in turn, were located more medial and ventral as compared to those produced by foot movements. Specifically, concerning mouth *goal-related* condition, the activations involved, bilaterally, the ventral part of the ventrolateral nucleus (VL), and, on the right, also the ventral part of the centro-median nucleus (CM; Suppl. Fig. 8 A). Concerning hand *goal-related* condition, the activations were found only on the left and involved VL, CM and the anteriormost part of the pulvinar; the activations in VL and CM extended more dorsally than those observed in mouth condition (Suppl. Fig. 8 B). In foot *goal-related* condition, the activations were observed only on the left, where they involved the lateral part of VL and ventro-lateral posterior nucleus (VPL; Suppl. Figure 8 C).


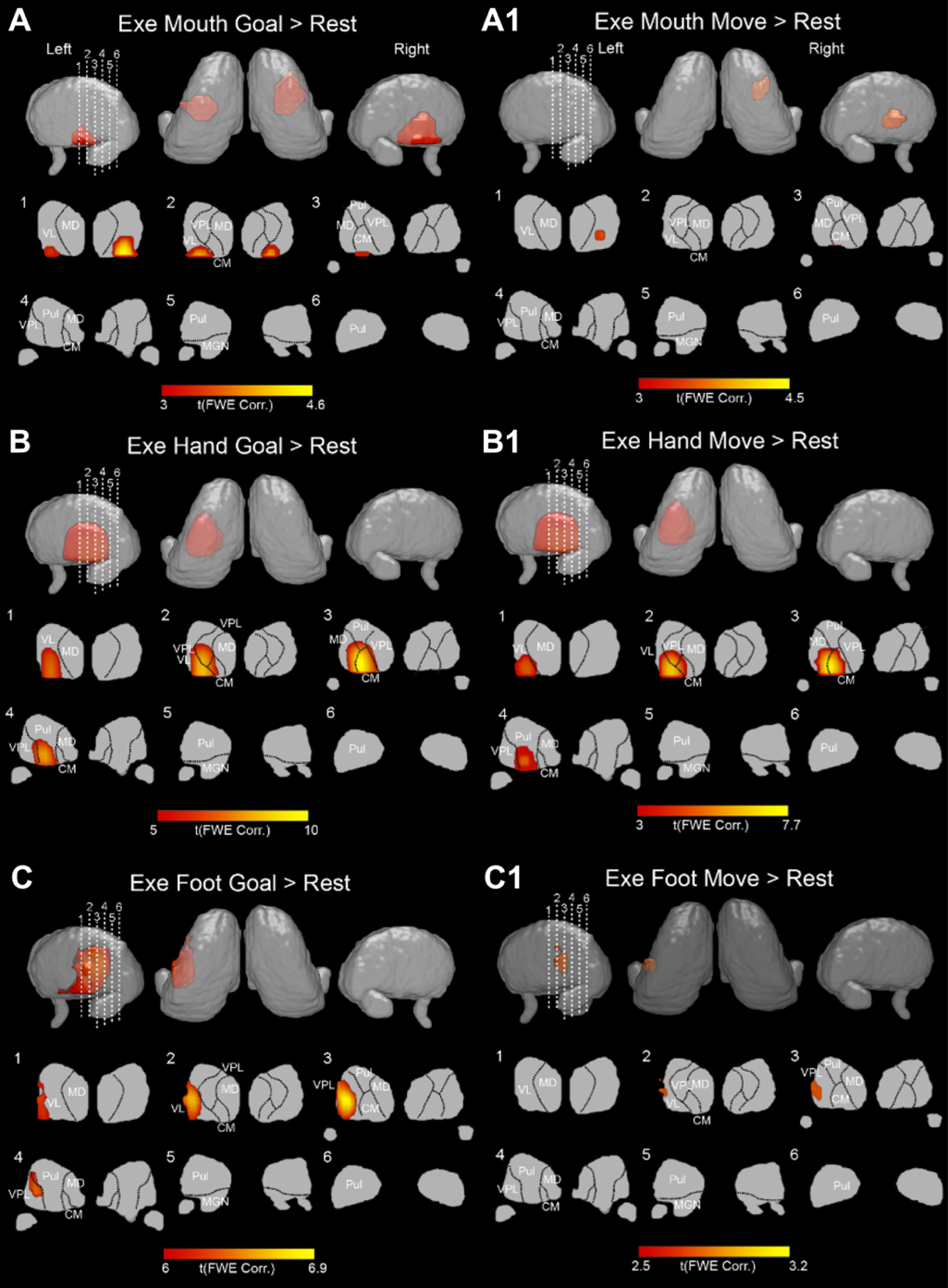


**Supplementary Figure 8.** Thalamic activations related to the contrasts *Exe Mouth Goal* > *Rest* (**A**), *Exe* *Mouth Move* > *Rest* (**A1**), *Exe Hand Goal* > *Rest* (**B**); *Exe Hand Move* > *Rest* (**B1**); *Exe Foot Goal* > *Rest* (**C**) and *Exe Foot Move* > *Rest* (**C1**). Thalamic activations are shown on 3D template (Thomas Atlas) (Su et al., 2019); left view, right view and axial view) and six coronal representative sections from ch2 template. Statistical threshold set at *p*<0.001 (FWE corrected at cluster level).

The activations observed in *simple movements* mouth and foot conditions were similar but less extensive than those observed in the corresponding *goal-related* ones. In particular, concerning mouth, the activations were found only in the ventral part of right VL while, concerning foot, they were located on lateral parts of left VL and VPL (Suppl. Fig. 8 A1 and C1). Concerning hand *simple movement* condition, the activations were very similar to those observed in the *goal-related* one, both for their extent and location, thus involving, only in left thalamus, the VL and CM nuclei and anterior part of the pulvinar (Suppl. Fig. 8 B1).

The thalamic activations found during the contrast *Obs* > *Rest* in all *goal-related* conditions are similar, independently of the considered effector, without showing any somatotopic organization. Specifically, the observation of all types of *goal-related* actions produced an extensive activation, especially in the pulvinar, that is bilaterally activated in its ventral part for almost the entire extent (Suppl. Fig. 9, A, B, and C). The activated voxels also extended bilaterally to the ventral part of VPL and, on the left, to CM. The activations found during the contrast *Obs* > *Rest* in *simple movements* conditions were similar but more restricted than those observed in *goal-related* ones, involving exclusively the pulvinar, bilaterally, for hand and foot and, in addition to it, a restricted part of left VPL and left CM, for the mouth (Suppl. Fig. 9, A1, B1, and C1).


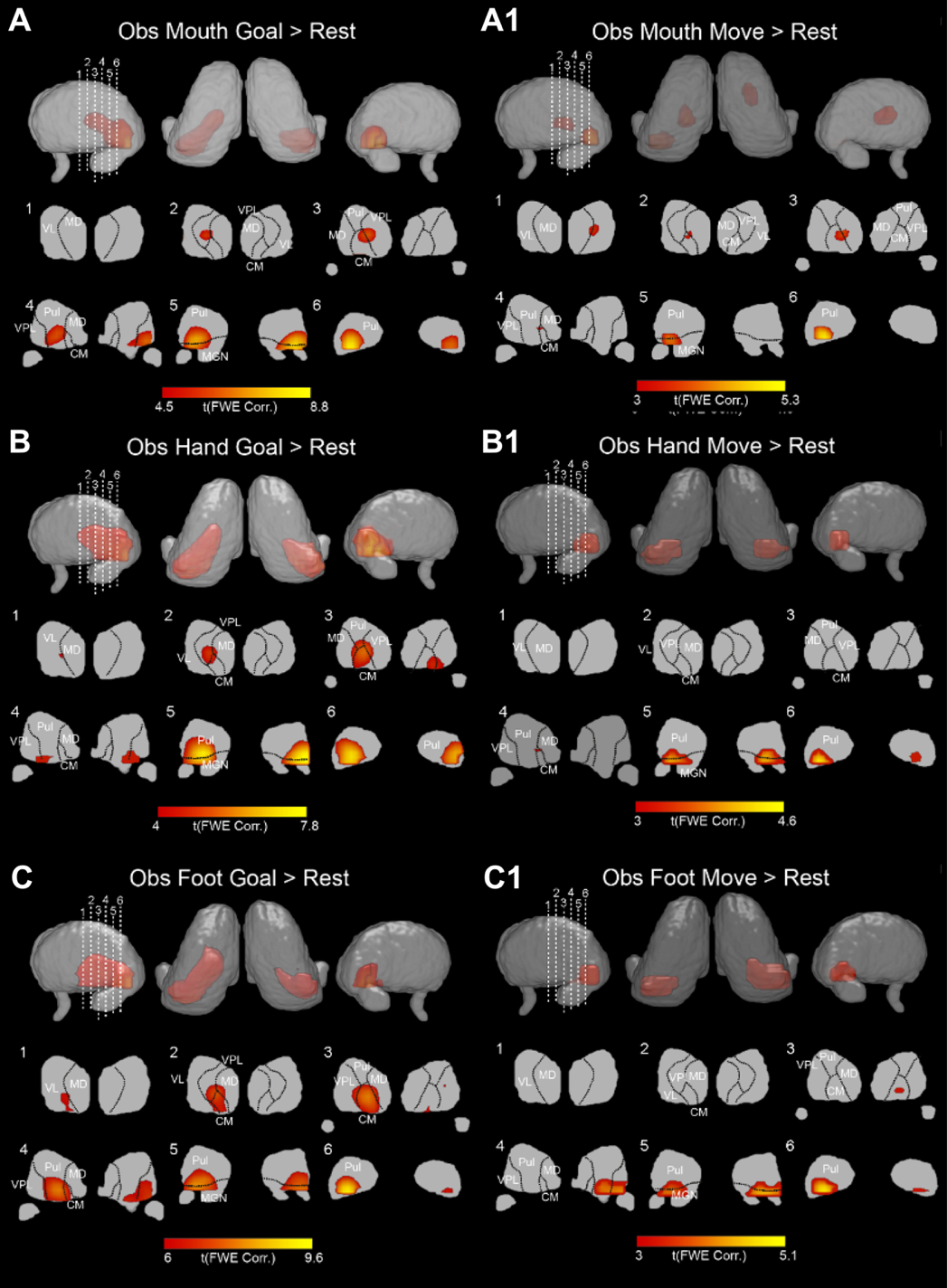


**Supplementary Figure 9.** Thalamic activations related to the contrasts *Obs Mouth Goal* > *Rest* (**A**), *Obs Mouth* *Move* > *Rest* (**A1**), *Obs Hand Goal* > *Rest* (**B**); *Obs Hand Move* > *Rest* (**B1**); *Obs Foot Goal* > *Rest* (**C**) and *Obs Foot Move* > *Rest* (**C1**). All activations are rendered with a threshold of *p*< 0.001 (FWE corrected at cluster level).

**
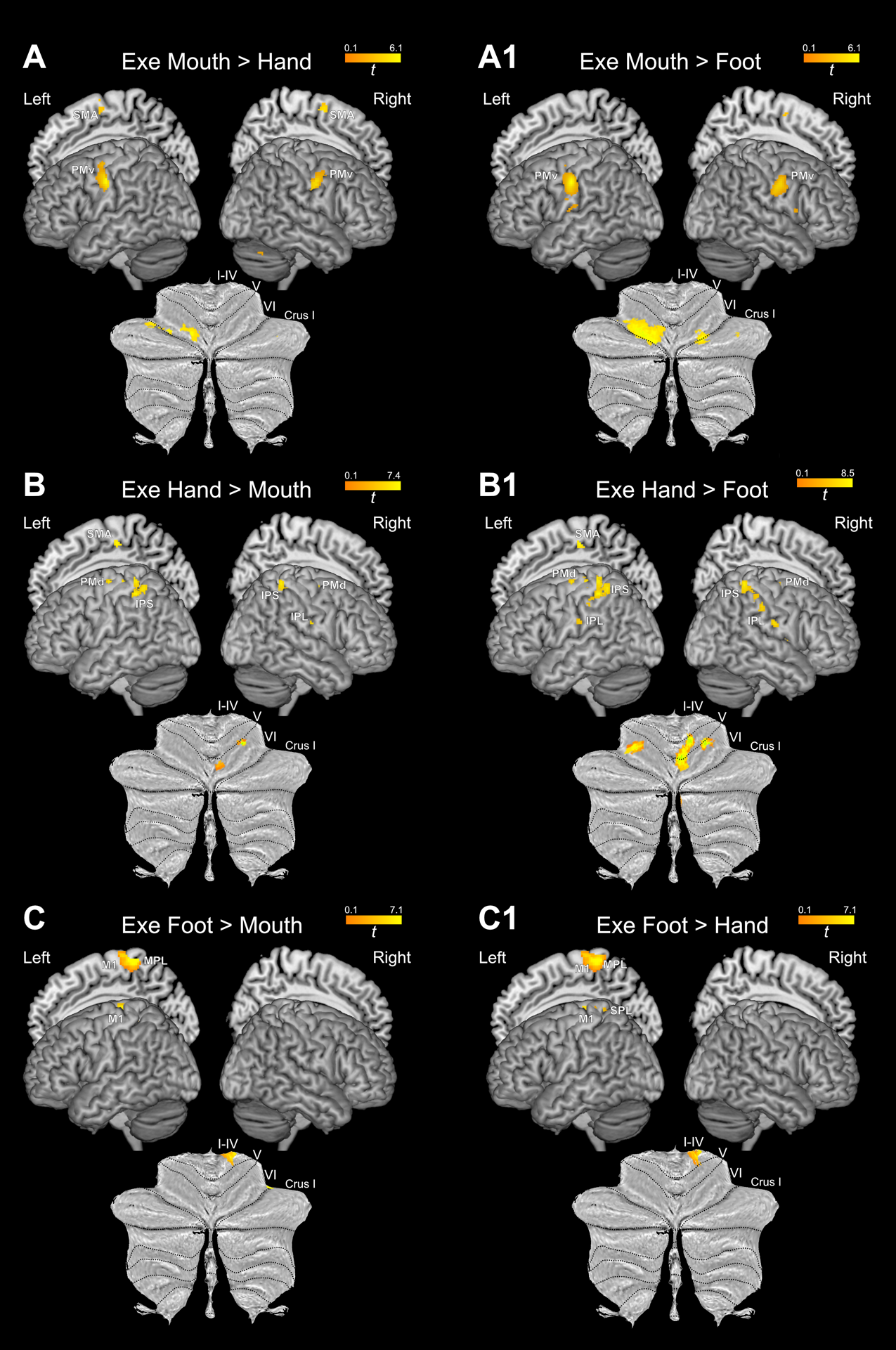
**

**Supplementary Figure 10.** Cortical and cerebellar activations resulting from the direct contrasts *between couples of effectors* during execution of actions vs movements (Goal > Move). (**A**) *Exe Mouth (Goal > Move) > Hand (Goal* > *Move)*, (**A1**) *Exe Mouth (Goal > Move) > Foot (Goal* > *Move)*, (**B**) *Exe Hand (Goal > Move) > Mouth (Goal* > *Move)*; (**B1**) *Exe Hand (Goal > Move) > Foot (Goal* > *Move)*; (**C**) *Exe Foot (Goal > Move) > Mouth (Goal* > *Move)* and (**C1**)*Exe Foot (Goal > Move) > Hand (Goal* > *Move)*. All contrast maps are rendered with a threshold of *p*<0.001 (FWE corrected at cluster level).
